# On maternity and the stronger immune response in women

**DOI:** 10.1038/s41467-022-32569-6

**Published:** 2022-08-18

**Authors:** Evan Mitchell, Andrea L. Graham, Francisco Úbeda, Geoff Wild

**Affiliations:** 1 Department of Mathematics, Western University, London, ON N6A 5B7 Canada; 2 Department of Ecology & Evolutionary Biology, Princeton University, Princeton, NJ 08544 USA; 3 Department of Biological Sciences, Royal Holloway, University of London Egham, Surrey, TW20 0EX United Kingdom

**Keywords:** Evolutionary theory, Coevolution, Computational models

## Abstract

Medical research reports that women often exhibit stronger immune responses than men, while pathogens tend to be more virulent in men. Current explanations cannot account for this pattern, creating an obstacle for our understanding of infectious-disease outcomes and the incidence of autoimmune diseases. We offer an alternative explanation that relies on a fundamental difference between the sexes: maternity and the opportunities it creates for transmission of pathogens from mother to child (vertical transmission). Our explanation relies on a mathematical model of the co-evolution of host immunocompetence and pathogen virulence. Here, we show that when there is sufficient vertical transmission co-evolution leads women to defend strongly against temperate pathogens and men to defend weakly against aggressive pathogens, in keeping with medical observations. From a more applied perspective, we argue that limiting vertical transmission of infections would alleviate the disproportionate incidence of autoimmune diseases in women over evolutionary time.

## Abstract (web summary)

Women generally mount a stronger immune response to infections than men do, resulting in a higher impact of autoimmune diseases. Here, the authors show that pathogen transmission from mother-to-child during pregnancy drives the co-evolution of a stout defence against harmless pathogens in women.

## Introduction

Many aspects of infections and immunity differ between women and men. For example, infections tend to be more frequent in men, immune responses are typically stronger in women (with women usually clearing infections faster), and pathogens are often more virulent in men (with men experiencing more severe outcomes)^1–6^. Many of the sex differences in the frequency, duration and severity of infectious diseases are attributable to differences in both the innate and the adaptive immune systems^1,3,6^. The innate immune system is always active, providing a fast and general response to infections^1,3,6^. The adaptive immune system is activated by the innate system and pathogen’s exposure, providing specific responses to infection^1,3,6^. Although there are complexities across the lifespan and among cell types^3^, both innate and adaptive immune responses are typically heightened in women, with higher numbers of macrophages and B-cells and greater activation of T and B-cells^1–5^. The higher number and activation of immune cells tend to result in faster clearance of infections and increased efficacy of vaccines in women, but also in their increased exposure to autoimmune disease^1–5^. Henceforth we use the term stronger immune system to refer to the faster clearance of infections resulting in a greater rate of recovery in women^3,6^. To date, the reasons why women should have a stronger immune system are not well understood^4,5,7^. Notice that we will focus on sex differences rather than gender differences (see ref. 8 for a discussion on sex and gender in immunity).

From a medical perspective, understanding the difference between the sexes in their immune function and response is critical to implementing treatments that are effective in both women and men. Furthermore, a strong immune system may result in immune cells attacking other cells of the same organism and causing autoimmune diseases^9–11^. By contrast, a weak immune system results in longer infections where pathogens may have a greater opportunity to disrupt the epigenome of host cells, causing cancer^12,13^. Understanding the differences in immune response between women and men is thus fundamental to address the disproportionate amount of autoimmune diseases affecting women and the disproportionate frequency of cancers affecting men^9–12^.

**Fig. 1:**
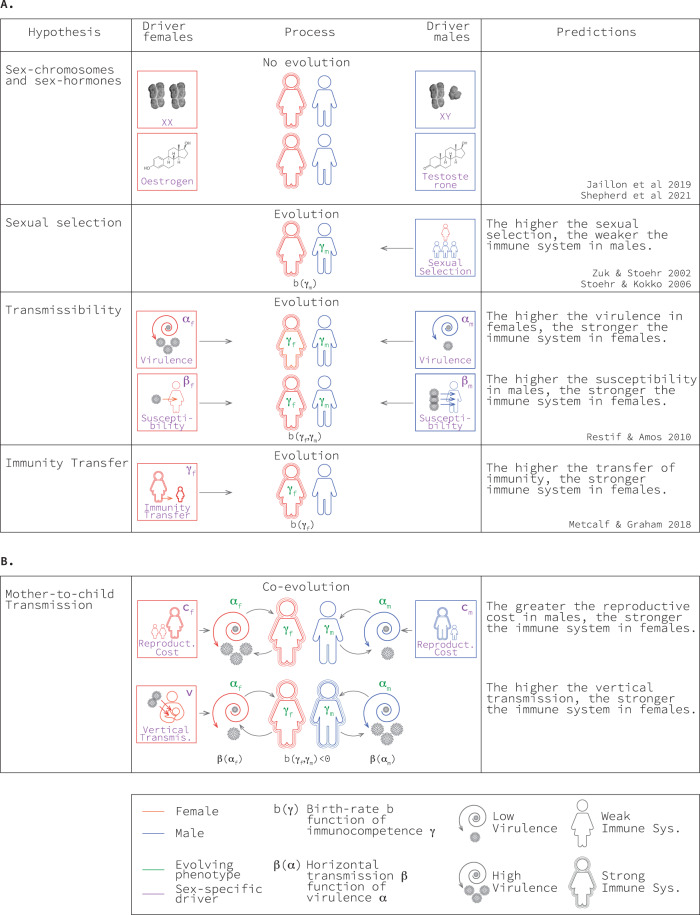
Hypotheses explaining stronger immunocompetence in women. Summary of the phenotypes evolving (in green), the drivers of their evolution (in purple), and the key predictions of each hypothesis. Panel **A** corresponds to previous work, while panel **B** corresponds to the work presented here.

Historically, it has been argued that the drivers of a stronger immune system in women are proximate physiological mechanisms^1–6,14^. In particular, previous work contends that sex hormones and sex chromosomes are the two main sources of sex-related asymmetry in immunocompetence. Sex hormones (progesterone, oestrogen and testosterone) play a role in the regulation of the immune system, with oestrogen enhancing immunocompetence and testosterone inhibiting it^1–6,14^. Because women exhibit higher levels of oestrogen and men exhibit higher levels of testosterone, differences in sex hormones could explain the mechanism underpinning a stronger immune system in women^1–6,14^. In addition, sex chromosomes provide women with two potentially different copies of X-linked genes and men with one copy of X-linked genes and Y-linked genes. Random X-chromosome inactivation in women results in cell mosaicism and incomplete inactivation may result in higher expression of specific genes in women. Y-linked genes provide a set of genes expressed only in men. Together, X-chromosome inactivation and/or escape from inactivation in women and Y-linked genes in men may explain the stronger immune system in women^1–6,14^ (Fig. 1).

Although proximate mechanisms for sex differences in immunocompetence have been proposed, there is relatively little research exploring the reasons why differences between the sexes in the strength of their immune systems (whether implemented by any means) may be advantageous for women and men. The scarcity of ultimate explanations is striking, as the immune system has direct effects on the fitness of individuals and is under strong selection^1,15^. We are aware of only three hypotheses aimed at explaining why women may have evolved a stronger immune system.

The first theory, henceforth the ‘sexual selection theory,’ considers the role of male competition for mating partners. This theory claims that testosterone, on the one hand, inhibits the immune system of males but, on the other hand, enhances their ability to compete for mating partners^16–19^. This theory predicts that when males are under strong competition for partners, natural selection favours greater investment in mating success, even when this comes at the cost of a weaker immune system^16–19^. Sexual selection thus favours the evolution of a weaker immune system in males as a result of the fitness costs associated with immune function in this sex (Fig. 1).

The second theory, henceforth the ‘transmissibility theory,’ considers the role of the pathogen’s ability to be transmitted to unrelated hosts (horizontal transmission). The difference between the sexes is introduced either by sex-related differences in susceptibility or sex-related differences in the pathogen’s ability to produce more copies of itself once established in its host (virulence). This theory predicts that when pathogens are more likely to infect males, natural selection favours a stronger immune system in females to clear infections faster^20^. It also predicts that when pathogens are more virulent in females, natural selection favours a stronger immune system in females^20^ (Fig. 1). The latter prediction of this model, however, is not consistent with the empirical observation that pathogens are more virulent in males.

A recent theory, henceforth the ‘immunity transfer theory,’ considers the role of maternal transference of immunity to her offspring. This theory predicts that when females can confer immunity to their offspring against horizontally transmitted pathogens, natural selection favours a stronger immune system in females, manifested as heightened detection of pathogens leading to faster clearance^21^ (Fig. 1).

**Fig. 2:**
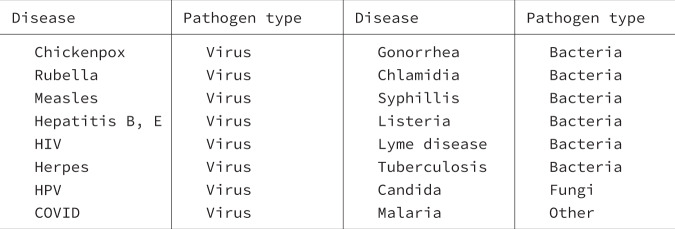
Examples of vertically transmitted diseases. Here we provide a non-exhaustive list of pathogens that are vertically transmitted.

Previous work does not consider the role of transmission of infections from mother to child on the evolution of women’s immune system. To refer to this transmission, in this research, we will use the terms ‘vertical transmission’ and ‘mother-to-child transmission’ interchangeably. Maternity, the period of time between conception and weaning, is a key life-history feature of females as far as natural selection is concerned. Success in conception and pregnancy is conditioned by a woman’s immune system^1^. Oestrogen and progesterone—the predominant female sexual hormones—by enhancing the immune system, not only prevent infections but also reduce fertility. This reduction in fertility can be caused by oestrogen-enhancing immune attacks on sperm, thus reducing the likelihood of conception and/or enhancing immune attacks on foetus resulting in miscarriage^1,22–25^. Furthermore, pregnancy and lactation open up a route for the transmission of pathogens that is unique to women^26^. During pregnancy and delivery, offspring can be born infected because some pathogens can cross the placenta and others enter the offspring at delivery^26^. Similarly, during lactation, offspring can be infected through breast milk^26^. Mother-to-child transmission is common in many infectious diseases (see examples in Fig. 2).

In addition, all previous work on the evolution of women’s immunocompetence provides a partial view of the evolutionary interaction between hosts and pathogens; a view that focuses on the evolution of hosts, ignoring the evolutionary feedback of pathogens^18,20^. It is well-known, however, that the evolution of hosts cannot be separated from the evolution of pathogens^18,27,28^. Therefore, it is critical to consider that the immunocompetence of hosts and the virulence of pathogens can simultaneously evolve in response to changes in one another (co-evolution).

Here, we explore whether the stronger immune system observed in women can be an adaptation of women to vertical transmission. For this purpose, we model the co-evolution between host immunocompetence and the pathogenic virulence exhibited by a pathogen. Because our approach is theoretical in nature, all findings we report require experimental support before being used to guide decisions regarding healthcare.

In our model, host immunocompetence is reflected in the rate of recovery from infection, *γ*_*i*_ with *i* = *f* for females and *i* = *m* for males. For its part, pathogen virulence is represented as the increased mortality rate experienced by those infected, *α*_*i*_ with *i* = *f* for female hosts and *i* = *m* for male hosts. We incorporate vertical transmission by assuming that infected mothers transmit the pathogen, at birth, to their offspring with probability *v* (i.e. the ‘vertical transmission rate’). Finally, to ensure that our findings can be compared to previous theory, we treat immunocompetence as a costly trait, with *c*_*i*_ representing the reproductive fitness cost paid by hosts of sex *i* = *f*, *m*.

Our work explains why women have evolved to recover more quickly from infections while pathogens have evolved a greater virulence in males. To make our case, we rely on a fundamental biological difference between the sexes, namely mother-to-child transmission. Our work challenges the pre-existing idea that vertical transmission reduces pathogen virulence in all cases^29,30^. We advance existing theory by providing a framework to study the co-evolution between immunocompetence and virulence in women and men when there is both horizontal and vertical transmission. More broadly, we consider the implications of our work for public health policies. In particular, we consider the efforts directed at reducing mother-to-child transmission on the immune systems of women and men and the disproportionate incidence of autoimmune disease in women and cancer in men.

## Results

### Equal cost for sexes and no mother-to-child transmission

**Fig. 3:**
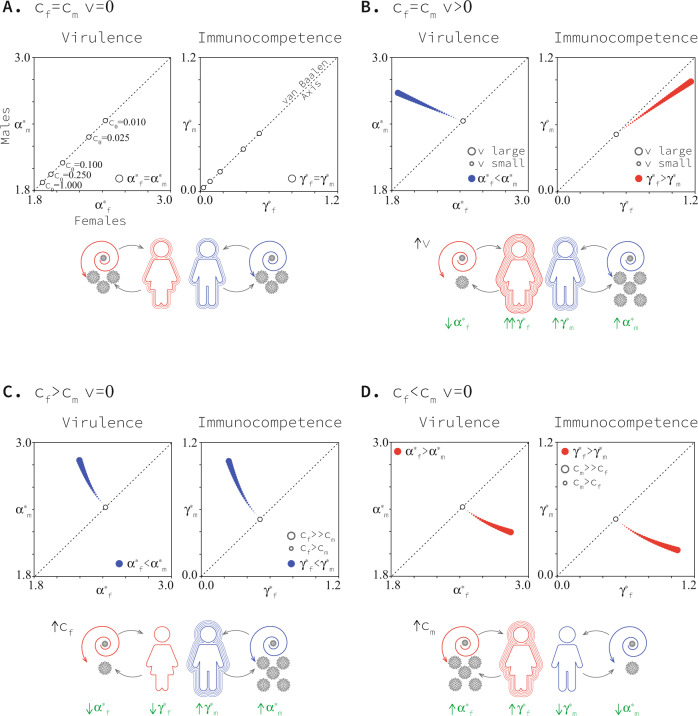
Co-evolved levels of virulence and immunocompetence in each sex. Panel **A** presents the case when there are no differences between the sexes. In the absence of differences between the sexes, trajectories converge to the main diagonal (the van Baalen axis), recovering previous non-sex-specific co-evolutionary results^27^. Panel **B** presents the case when the only difference between the sexes is the mother-to-child transmission rate, that is *v* > 0 and *c*_*f*_ = *c*_*m*_. Panels **C**, **D** present the case when the only difference between the sexes is the fitness cost of immunocompetence, that is *c*_*f*_ ≠ *c*_*m*_ and *v* = 0. Results are based on *c*_0_ = 0.01 (panels **B–D**), *b*_max_ = 2, *β*_*max*_ = 1.1, *d* = 4, μ = 0.1, and *v* ranges between 0 and 0.9. See Methods for an explanation of model parameters. Source data are provided as a Source Data file.

We first assume mothers do not transmit pathogens to their children (*v* = 0) to establish a baseline set of model predictions. In this case, if the fitness cost of recovery from infection for females is the same as the fitness cost of recovery for males (*c*_*f*_ = *c*_*m*_) then selection favours defended hosts and virulent parasites, in agreement with the previous work^27^. Furthermore, selection leads to co-evolved levels of immunocompetence and virulence (i.e., ${\gamma }_{i}\to {\gamma }_{i}^{*}$ and ${\alpha }_{i}\to {\alpha }_{i}^{*}$ over time) that are independent of the sex of the host (in the van Baalen axis of symmetry; Fig. 3A).

### Unequal costs in the sexes and no mother-to-child transmission

When mothers do not transmit pathogens to their children (*v* = 0) and the cost of recovery is not equal in the sexes (*c*_*f*_ ≠ *c*_*m*_), it is the sex-specific costs that determine the qualitative outcome of the co-evolutionary process described by our model. The sex that pays the lower cost of recovery can better afford a higher level of immune function. This sets the stage for higher virulence of pathogens infecting hosts of that sex, as pathogens compensate for reduced residency time within a host by increasing their rate of horizontal transmission between hosts, and increased virulence is, by the assumptions of our model, an inevitable consequence of this compensation (Fig. 3C, D). In this case, sex-specific costs of recovery lead to the co-evolution of levels of immunocompetence and virulence that are themselves sex-specific (away from the diagonal in Fig. 3C, D). The predicted pattern is one in which both immunocompetence and virulence are stronger in the same sex, that is, both stronger in females or both stronger in males (Fig. 3C, D).

More specifically, when the cost disparity between the sexes is increased, while keeping the midpoint cost (*c*_*f*_ + *c*_*m*_)/2 = *c*_0_ fixed, we find (i) the resulting co-evolved immunocompetence is enhanced in the sex with the cheaper immune system and lowered in the sex with the more expensive system and (ii) the co-evolved virulence is enhanced in the sex with the cheaper immune system and lowered in the sex with the more expensive immune system (Fig. 3C, D).

Our results show that when protection against disease is more expensive for females, their immunocompetence will be weaker and pathogens will exhibit higher virulence when infecting males, i.e., ${\gamma }_{m}^{*} \, > \, {\gamma }_{f}^{*}$ and ${\alpha }_{m}^{*} \, > \, {\alpha }_{f}^{*}$ (Fig. 3C). Similarly, when protection against disease is more expensive for males, their immunocompetence will be weaker relative to females and pathogens will exhibit higher virulence when infecting females, i.e., ${\gamma }_{m}^{*} \, < \, {\gamma }_{f}^{*}$ and ${\alpha }_{m}^{*} \, < \, {\alpha }_{f}^{*}$ (Fig. 3D).

### Equal cost for sexes and mother-to-child transmission

When mothers transmit pathogens to their children (*v* > 0) and the cost of recovery is equal in both sexes (*c*_*f*_ = *c*_*m*_), the likelihood of mother-to-child transmission determines the qualitative outcome of the co-evolutionary process described by our model. Specifically, low mother-to-child transmission leads to stronger immunocompetence in females (Fig. 3B) in all cases. High mother-to-child transmission, however, can sometimes lead to stronger immunocompetence in females (Fig. 3B), but can, at other times, lead to stronger immunocompetence in males, depending on the life-history trade-off faced by the pathogen (Fig. 5, see Methods and Supplemental Material). In all cases, there is higher virulence of pathogens infecting males (Fig. 3B). Mother-to-child transmission also leads to the evolution of levels of immunocompetence and virulence that are sex-specific (away from the van Baalen axis of symmetry; Fig. 3B). Mother-to-child transmission allows for a pattern in which females exhibit stronger immunocompetence compared to males while pathogens are more virulent when infecting males as opposed to females (Fig. 3B).

**Fig. 4:**
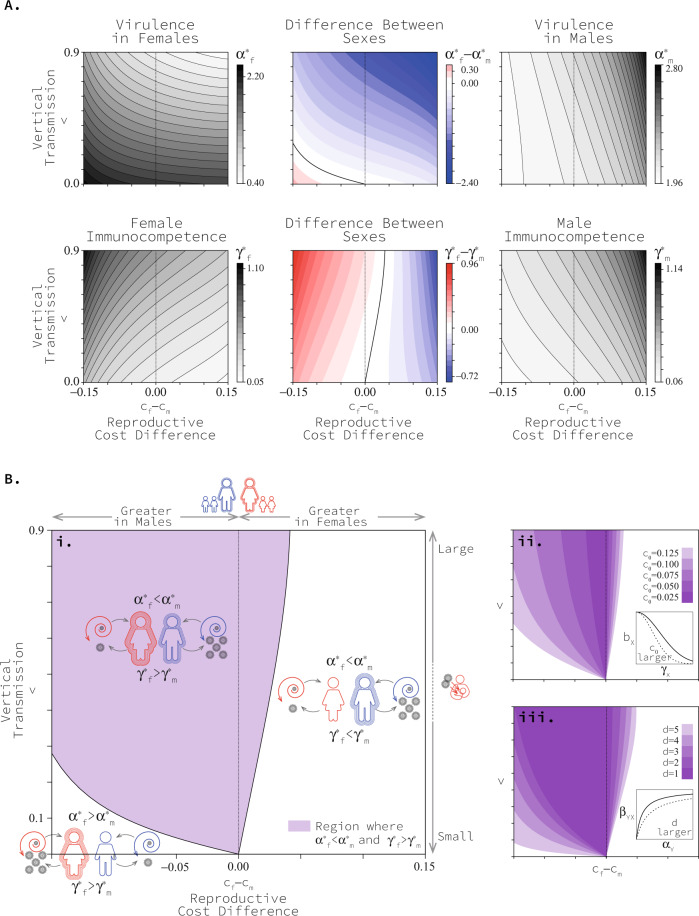
Co-evolved levels of virulence and immunocompetence in each sex as vertical transmission and difference in the cost of immune function changes. In panel **A** black and white sub-panels present the virulence and immunocompetence levels in each of the sexes, while coloured centre sub-panels present the virulence and immunocompetence of the sexes relative to each other. In these sub-panels red areas correspond to greater values in females and blue areas correspond to greater values in males. Black curves in centre sub-panels correspond to zero differences between the sexes and separate red regions from blue ones. Panel **B.i** combines the black curves in panel (**A**) centre sub-panels to define three regions corresponding to the three qualitatively different co-evolutionary patterns. Panels **B.ii, iii** show how these regions change as parameter values affecting the trade-off in host *c*_0_ and pathogen *d* change. Results are based on *c*_0_ = 0.1, *b*_*max*_ = 2, *β*_*max*_ = 1.1, *d* = 4 and μ = 0.1. See Methods for an explanation of model parameters. Source data are provided as a Source Data file.

More specifically, we find that the co-evolved level of immunocompetence of both sexes grows along with the likelihood of mother-to-child transmission (Fig. 4A). In most cases (and always when the likelihood of mother-to-child transmission is low), the immunocompetence in females increases at a faster rate than it does in males (Fig. 4A). This leads to stronger immunocompetence of females (Fig. 3B). We find that the co-evolved virulence of pathogens infecting females is reduced as the likelihood of mother-to-child transmission grows, while that of pathogens infecting males increases (Fig. 4A). This leads to stronger virulence of pathogens infecting males (Fig. 3B). The latter result challenges the consensus in the field that vertical transmission universally reduces the virulence of pathogens^29,31^. Previous work assumes that pathogen’s virulence evolves independently from host’s immunocompetence^31^. We show that when virulence and immunocompetence co-evolve, vertical transmission can result in the evolution of higher virulence. Remarkably, it is vertical transmission, a trait in females, that leads to the evolution of higher virulence in males in this framework.

### Unequal costs for sexes and mother-to-child transmission

Considering both contributions of maternity—sex-specific costs of recovery and mother-to-child transmission—we identify the same three qualitatively distinct co-evolutionary outcomes revealed by the special cases above (Fig. 4). Each outcome can be mapped to a distinct region of parameter space (Fig. 4B).

First, we predict females recover more quickly and suffer higher virulence when the cost of recovery is higher in males and mother-to-child transmission is sufficiently low (Fig. 4B.i). Second, we predict males recover more quickly and suffer higher virulence when the cost of recovery is higher in females (Fig. 4B.i). Third, we predict females recover more quickly yet suffer lower virulence when the cost of recovery is higher in males and mother-to-child transmission is sufficiently high (Fig. 4B.i). How low or high vertical transmission has to be will change along with life-history details of hosts and pathogens (Fig. 4B.ii–iii).

In our model, we find a wide range of parameter values that support stronger female immunocompetence, represented by the rate of recovery among infected individuals of this sex. Furthermore, the range of parameter values where stronger female immunocompetence and high virulence in males are predicted is also significant. This pattern requires mother-to-child transmission but can be observed both when mother-to-child transmission is low (as long as the difference in reproductive cost is slightly greater in males) and high (for both, when reproductive costs are greater in males and when reproductive costs are greater in females) and for a wide range of parametrisations.

## Discussion

Our work finds that the stronger immune response observed in women^1–6,14^ can be understood as an evolutionary consequence of pathogen transmission from mothers to their children. We predict that, in the absence of any other differences between the sexes, even the smallest amount of vertical transmission results in faster recovery in women (Figs. 3B, 4). Furthermore, greater fitness costs of immunocompetence in men enhance this bias in immunocompetence toward women (Figs. 3D, 4). In this sense, we find that mother-to-child transmission and sexual selection work together, favouring greater immunocompetence in women. The transmission of pathogens from mothers to their children is a fundamental biological difference between the sexes. It is common during pregnancy when smaller pathogens can cross the placenta, and during natural delivery, when offspring swallow or inhale all types of pathogens^26^. In addition, during the lactation period, newborns can be infected through breast milk and close contact^26^. Vertical transmission has been reported in many infectious diseases, including malaria, tuberculosis, hepatitis and, most recently, COVID-19 (see Fig. 2 for a non-exhaustive list of diseases that are vertically transmitted).

We also find that mother-to-child transmission drives the co-evolution of higher virulence in men and a stronger immune response in women. Importantly, this combined pattern of virulence and immunocompetence is the one observed by medical researchers and cannot be explained by any of the existing hypotheses^1–6,14,17,21^. In the absence of any other difference between the sexes, we find that any small amount of mother-to-child transmission results in higher virulence in men and faster recovery in women (Fig. 4). Higher costs of immunocompetence in men alone cannot explain the combined pattern of higher virulence in men and stronger immunocompetence in women (Fig. 4). The sexual-selection, transmissibility, and immunity transfer hypotheses in their current formulations consider the evolution of host immunocompetence ignoring the feedback from co-evolving pathogens^18,20^. However, co-evolution between hosts and pathogens is crucial for a complete picture of the natural history of infectious diseases^27,28^. Our work not only considers previously unrecognised drivers for the evolution of stronger immunocompetence—vertical transmission and costs of immunocompetence in females—but does it in a broader co-evolutionary framework.

Our work only provides partial support for the sexual selection hypothesis, whereby the greater cost of immunocompetence in men leads to the evolution of greater immunocompetence in women. However, we find that in extending the tenets of the sexual selection hypothesis to a co-evolutionary framework, the emerging pattern is one of higher virulence and stronger immunocompetence in women, which is contrary to medical observations^1–6,14,17^.

Our model predicts that mother-to-child transmission of pathogens drives the co-evolution of a pattern where women put up a stouter defence against relatively harmless pathogens and men put up a weaker defence against fierce pathogens. Why fight harder when being exploited to a lesser degree? While this pattern may seem counterintuitive at first, a complete argument recognises that mother-to-child transmission introduces an extra cost of infection—a reduction in the quality of offspring produced—in addition to the ever-present threat of mortality. Females, and females alone, can modulate the extra cost of infection associated with mother-to-child transmission directly by adjusting their immunocompetence. Thus, females have an extra incentive to clear infections more readily than males, even when these infections are more virulent in males. Pathogens infecting females do not respond to the increased immunocompetence with substantial changes to their virulence because trans-generational transmission also acts as an evolutionary brake. An increase in virulence when infecting females would, of course, limit a pathogen’s ability to exploit mother-to-child transmission pathways. In summary, our model shows that mother-to-child transmission decouples the pattern of mutual escalation/de-escalation predicted by asymmetries in fitness costs of immunocompetence between the sexes (horizontal axis in Fig. 4B).

Because host immunocompetence impacts its reproductive success, the immunocompetence-virulence patterns discussed here apply to women and men of reproductive age. In post-reproductive stages, evolutionary forces may change. In principle, post-reproductive individuals could invest resources in immunocompetence without experiencing the cost of reduced fertility as they do not produce offspring anymore. The expected outcome would be the enhancement of cost-free immunocompetence by the host followed by the enhancement of virulence by the pathogen to compensate. In principle, an erosion of the differences in immunocompetence between the sexes in post-reproductive age would be expected, but due to the difference between the sexes in the time in which they cease to reproduce (earlier in women than men), one would expect that immunocompetence in women would be heightened following menopause well before men catch up. This would not be the case, however, if post-reproductive individuals contribute to the reproductive success of their offspring, as the ‘Grandmother Hypothesis’ argues in the context of the evolution of menopause (see refs. 32, 33 for the hypothesis and refs. 34, 35 for empirical support). If a grandmother provides assistance in raising her grand-offspring, she would still be trading immunocompetence for fertility although, this time, mediated by her grandchildren. Furthermore, due to close contact with her own grandchildren, she would be a source of generalised vertical transmission (generalised, in the sense that infection would potentially occur in a manner that was not sensitive to the prevalence of the disease in the broader population). In this scenario, our findings fully apply to post-reproductive men and women. A proper analysis of the change in evolutionary forces with the reproductive state would require a much complex model with age structure in the population. While this is beyond the scope of our paper, it is a promising avenue for future research.

Our results challenge the widely accepted notion that mother-to-child transmission should reduce virulence. Previous work explored the effect of vertical transmission on the evolution of virulence—ignoring host feedback—finding that vertical transmission reduces virulence in general^29,30^. When allowing for plasticity in the evolution of pathogen’s virulence, vertical transmission results in more tamed pathogens in women and has no effect in men^31^. Here we show that in a co-evolutionary context, vertical transmission leads to more tamed pathogens in women but more aggressive pathogens in men (Fig. 3B). The reason why is that vertical transmission enhances the immunocompetence in males—although to a lesser degree than it does in females—which leads to pathogens becoming more virulent to compensate. In the absence of sex-specific plasticity, this may result in parameter regions where virulence may increase with vertical transmission.

**Fig. 5:**
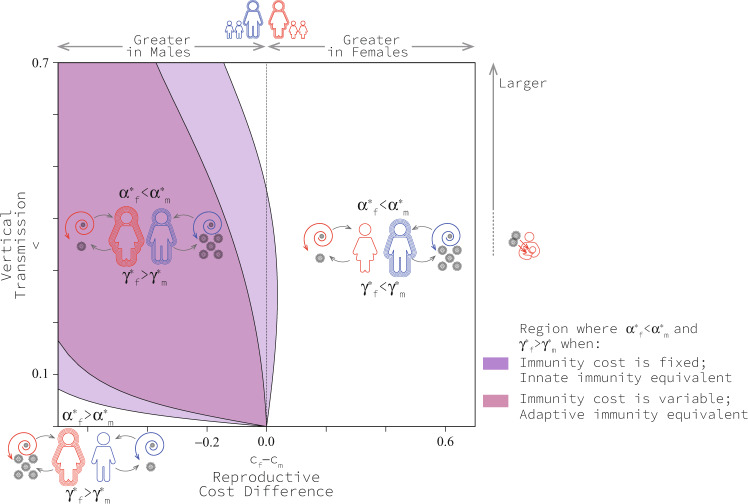
Regions of qualitatively different co-evolutionary patterns when the fitness cost of immune investment is fixed and variable. Fixed immunity costs are those experienced in all cases (with or without infection) and best reflect innate immunity costs. Variable immunity costs are those experienced with infection only and best reflect adaptive immunity costs. Results are based on *c*_0_ = 0.5, *b*_*max*_ = 3.5, *β*_*max*_ = 3.1, *d* = 4 and μ = 0.1. See Methods for an explanation of model parameters. Source data are provided as a Source Data file.

Higher virulence in men and stronger immunocompetence in women can be observed when we are modelling both innate or adaptive immunocompetence. Here, we assumed that the cost of an immune system was a cost experienced by the host both when it was infected and not infected. This corresponds to a cost of maintenance of the immune system, which relates best to the cost of innate immunocompetence. An alternative assumption (modelled in the Supplemental Material) is that the cost of an immune system is experienced only by infected hosts. This corresponds to the cost of activating the immune system in response to an infection which relates best to the cost of adaptive immunocompetence. We find that our results do not change qualitatively when modelling innate as opposed to adaptive immunocompetence (Fig. 5). The only qualitative difference is that the region of parameter space where the pattern we are interested in can be observed shrinks when the immunocompetence costs are constant (Fig. 5).

**Fig. 6:**
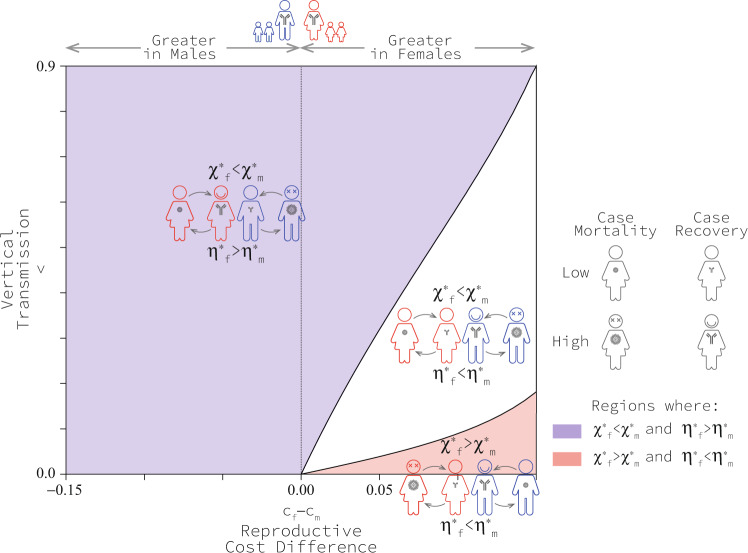
Regions of qualitatively different co-evolutionary patterns when measured as case mortality *χ*_*i*_ for *i* = *f*, *m*, and case recovery *η*_*i*_ for *i* = *f*, *m* as usually done in medical records. Results are based on *c*_0_ = 0.1, *b*_*max*_ = 2, *β*_*max*_ = 1.1, *d* = 4 and μ = 0.1. See Methods for an explanation of model parameters. Source data are provided as a Source Data file.

There are different ways of measuring virulence and immunocompetence^36^. In this research, we used the conventional definition of virulence as the disease-related mortality rate (*α*_*i*_), and we used the recovery rate (*γ*_*i*_) to reflect immunocompetence. However, medical researchers often measure the probability of an infected individual dying from an infection (‘case mortality,’ denoted here as *χ*_*i*_ for *i* = *f*, *m*) as virulence, and the probability of an infected individual recovering from infection (‘case recovery,’ denoted here as *η*_*i*_ for *i* = *f*, *m*) as a measure of immunocompetence^36^. If we change our thinking and measure virulence as the probability of mortality and immunocompetence as recovery in infected individuals, the range of scenarios in which women recover from infections more readily and men suffer virulent effects more strongly expands significantly. More to the point, when translating our results into these metrics, we observe that the region of interest where case mortality is high in males and case recovery is high in females occupies a larger portion of the parameter space (Fig. 6). Remarkably, this region extends to the horizontal axis where the cost of immunocompetence in men is greater than in women even if there is no mother-to-child transmission (Fig. 6).

From a more applied perspective, our results can help explain the higher incidence of autoimmune diseases in women and of cancers in men. A strong immune system to fight weak pathogens may result in the long-term development of autoimmune diseases. Our work predicts a higher incidence of autoimmune diseases in women. Evidence does show that the incidence of autoimmune diseases is much higher in women^9–11,37,38^. A longer time to clearance of pathogens replicating at high rates gives pathogens more time to modify the epigenetic signature of host cells in order to speed up cell replication. Such modifications to the epigenetic signature may result in the development of cancers in individuals that recovered from infections. Our work predicts a higher incidence of cancers in men as they take longer to clear infections. Evidence does show that the incidence of cancers is much higher in men^12^. Notice that susceptibility to autoimmune diseases in women increases with age^39^. This is consistent with our model as the fertility cost of investing in immunocompetence reduces with age. We thus would naively predict that immunocompetence would be enhanced with age and more in women than men, thus resulting in a greater incidence of autoimmune disease in older women. Notice, however, that this would need to be verified by extending our model to include age structure.

Our work suggests that the population-wide use of drugs (or other methods) to prevent mother-to-child transmission of infectious diseases will ultimately reduce the incidence of autoimmune disease in women and the virulence of infectious diseases in men over evolutionary time. Reduction of mother-to-child transmission is predicted to reduce the virulence of pathogens infecting men and the immunocompetence of women (Fig. 3). Therefore, public health policies aimed at reducing mother-to-child transmission may help to alleviate the disproportionate incidence of autoimmune disease in women^9–11,37,38^. The flip side of such policies is that they will increment the disproportionate incidence of cancer in men^12^ as our work suggests that they would weaken their immunocompetence (Fig. 3).

In general, a better understanding of what causes the observed differences in immunocompetence between women and men should bring us closer to implementing sex-specific medical treatment, thus improving therapeutic choices. Differences between the sexes with respect to infectious diseases have been well documented since the 1960s and can be reflected in the immune response itself or the rate of response to drug-based treatments^40–42^. Instead of controlling for differences between the sexes, the reaction of scientists was to exclude women from clinical trials or ignore sex differences in their analyses^43^. It is hardly surprising, therefore, that women are now 50% more likely to develop an adverse reaction to a therapeutic drug than men are^40–42^. Scientists have begun to account for sex differences only in the last decade, often in response to pressure from funding bodies^40–42^. Our hope is that this work brings further attention to the need to account for sex in epidemiological research to better understand the effects of public-health measures for everyone.

## Methods

### Ecological model

We start with an ecological model of resident host-pathogen dynamics that assumes these populations are, respectively, genetically homogeneous. The ecological model underlies the evolutionary model we develop later. A complete description of the model, and the methods of analysis that follow, can be found in the Supplementary Information.

We consider a population of hosts classified according to their sex and disease status. At time *t*, there are *S*_*i*_ = *S*_*i*_(*t*) sex-*i* individuals not infected by the pathogen, but susceptible to future infection (*i* = *f* for females, *i* = *m* for males). At time *t* there are also *I*_*i*_ = *I*_*i*_(*t*) sex-*i* individuals who are not only infected with the pathogen but also able to transmit their infection to others. Our specific goal in this section is to develop a mathematical description of how the numbers of hosts in the various classes change over time.

**Fig. 7:**
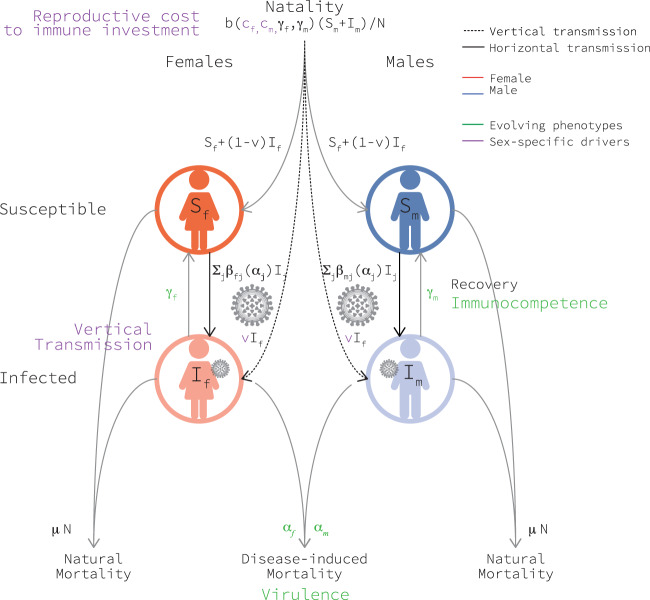
Compartmental diagram of the ecological model upon which our co-evolutionary model is based. This model incorporates two sexes (females in red and males in blue) and vertical transmission (dashed line). The flow between compartments is represented by arrows and expressions next to each arrow represent the flow rate. Evolving phenotypes and drivers of their evolution are indicated in green and purple, respectively. Source data are provided as a Source Data file.

The number of hosts in the population changes as a result of birth events. Following previous work^44,45^, we model the host mating rate using the harmonic mean of the population sizes of females and males. Assuming a one-to-one birth sex ratio, then newly born hosts of either sex join the population at rate $\frac{b({S}_{f}+{I}_{f})({S}_{m}+{I}_{m})}{N}$ where *b* > 0, and *N* = *N*(*t*) denotes the total population size at time *t*. We assume that newborns produced by susceptible mothers are, themselves, susceptible. By contrast, we suppose that newborns produced by infected mothers acquire their mother’s infection with probability *v*, where *v* is what we have called the ‘vertical transmission rate’^31^. Host number also changes because of death events. Hosts in every class experience natural mortality at per-capita rate *μN*, where *μ* is a positive constant. Hosts infected by the pathogen also experience disease-related mortality at per-capita rate *α*_*i*_ (a measure of pathogen ‘virulence’) (Fig. 7).

Numbers of hosts in any particular class changes as their disease-status changes. For example, we allow infected individuals to recover at per-capita rate *γ*_*i*_ (a measure of host ‘immunocompetence’). We assume that, upon recovery, hosts move immediately into the appropriate susceptible group. In this way, we ignore the possibility that recovery implies immunity to subsequent infection. The disease status of hosts can also change because of horizontal disease-transmission events. We approach horizontal transmission in a standard way and assume that susceptible sex-*i* hosts acquire the pathogen horizontally from their infected sex-*j* counterparts at a total rate of *S*_*i*_*β*_*ij*_*I*_*j*_. Here, *β*_*ij*_ is a constant that reflects the transmissibility of the pathogen. We assume that when a host acquires an infection horizontally, it immediately becomes infectious (Fig. 7).

The model described above is summarised mathematically using the following system of differential equations:

1a $$\frac{d{S}_{f}}{dt}=\frac{b({S}_{f}+(1-v){I}_{f})({S}_{m}+{I}_{m})}{N}+{\gamma }_{f}{I}_{f}-{S}_{f}{\beta }_{ff}{I}_{f}-{S}_{f}{\beta }_{fm}{I}_{m}-\mu N{S}_{f}$$

1b $$\frac{d{S}_{m}}{dt}=\frac{b({S}_{f}+(1-v){I}_{f})({S}_{m}+{I}_{m})}{N}+{\gamma }_{m}{I}_{m}-{S}_{m}{\beta }_{mf}{I}_{f}-{S}_{m}{\beta }_{mm}{I}_{m}-\mu N{S}_{m}$$

1c $$\frac{d{I}_{f}}{dt}=\frac{bv{I}_{f}({S}_{m}+{I}_{m})}{N}+{S}_{f}{\beta }_{ff}{I}_{f}+{S}_{f}{\beta }_{fm}{I}_{m}-({\gamma }_{f}+{\alpha }_{f}+\mu N){I}_{f}$$

1d $$\frac{d{I}_{m}}{dt}=\frac{bv{I}_{f}({S}_{m}+{I}_{m})}{N}+{S}_{m}{\beta }_{mf}{I}_{f}+{S}_{m}{\beta }_{mm}{I}_{m}-({\gamma }_{m}+{\alpha }_{m}+\mu N){I}_{m}.$$

Under a reasonable set of conditions, the previous system tends, over time, to an equilibrium state in which infections are endemic.

### Co-evolutionary model

To study how pathogen’s disease-induced mortality and the host’s immune system respond to selection, we assume that each faces a life-history trade-off.

First, the pathogen’s ability to transmit horizontally trades off against the duration of any given infection it establishes. Following the previous authors^30,46,47^, we capture this trade-off by assuming

2 $${\beta }_{ij}=\beta ({\alpha }_{j})=\frac{{\beta }_{\max }{\alpha }_{j}}{{\alpha }_{j}+d}\quad \,{{\mbox{for}}}\,\,j=f,\;m,$$

where ${\beta }_{\max },\,d \, > \, 0$ are constants. Equation (2) implies that the nature of the trade-off faced by a pathogen is the same in both female and male hosts. Specifically, a pathogen can only increase its rate of horizontal transmission by increasing the disease-induced mortality rate experienced by its host (which, in turn, reduces the duration of infection). Equation (2) also says the horizontal transmission rate saturates at ${\beta }_{\max }$ (independent of host sex), and does so more quickly as the parameter *d* is reduced (again, independent of host sex). Note also that Equation (2) does not depend on *i*: the sex of the susceptible host to whom the pathogen is transmitted.

For their part, hosts face a trade-off between investing resources in their immune system and their reproductive success. Increased immune investment is reflected in an increased recovery rate. To capture the host’s trade-off, then, we treat birth rate *b* as a decreasing function of the recovery rate. Moreover, we assume that the decrease in *b* is experienced by the host regardless of its disease status. In other words, we assume that cost associated with the immune system is an ongoing one, incurred mainly because of maintenance^27^ (this assumption model innate immunocompetence best) rather than being due to the activation that follows an infection^48^ (this assumption would model adaptive immunocompetence best). As noted in the Discussion, we relax this assumption in the Supplemental Material and compare the results for maintenance and activation costs. As an example, here, we point to evidence that shows female sex hormones enhance the immune system but simultaneously reduce the likelihood of conception and increase the chances of spontaneous abortion^49–51^. In mathematical terms, we capture the host’s trade-off using

3 $$b=b({\gamma }_{f},{\gamma }_{m})={b}_{\max }\,{e}^{-{c}_{f}{\gamma }_{f}^{2}}\,{e}^{-{c}_{m}{\gamma }_{m}^{2}}$$

where *c*_*i*_ reflects the rate at which fertility is reduced as sex-*i* immune function is increased (‘cost of recovery’ above). Equation (3) generalises the birth rate functions used previously^27,48^ to our sex-specific setting. The fact that *b* in this equation depends on both *γ*_*f*_ and *γ*_*m*_ reflects the fact that the reduced fertility of one mate affects the fertility of its partner^16^.

Our approach to modelling the co-evolution of host and pathogen is rooted in the adaptive-dynamics methodology^52–54^. For the pathogen population, we build a fitness expression that measures the success of a rare mutant strain in a population close to the endemic equilibrium established by the system (1) (indicated as ${\bar{S}}_{i}$, ${\bar{I}}_{i}$, and $\bar{N}$). Assuming that the mutant strain of pathogen is associated with a disease-induced mortality rate equal to ${\tilde{\alpha }}_{i}$ in sex-*i* hosts, the number of mutant infections, ${\tilde{I}}_{i}={\tilde{I}}_{i}(t)$ changes according to

4a $$\frac{d{\tilde{I}}_{f}}{dt}=\frac{bv{\tilde{I}}_{f}({\bar{S}}_{m}+{\bar{I}}_{m})}{\bar{N}}+{\bar{S}}_{f}\beta ({\tilde{\alpha }}_{f}){\tilde{I}}_{f}+{\bar{S}}_{f}\beta ({\tilde{\alpha }}_{m}){\tilde{I}}_{m}-({\gamma }_{f}+{\tilde{\alpha }}_{f}+\mu \bar{N}){\tilde{I}}_{f}$$

4b $$\frac{d{\tilde{I}}_{m}}{dt}=\frac{bv{\tilde{I}}_{f}({\bar{S}}_{m}+{\bar{I}}_{m})}{\bar{N}}+{\bar{S}}_{m}\beta ({\tilde{\alpha }}_{f}){\tilde{I}}_{f}+{\bar{S}}_{m}\beta ({\tilde{\alpha }}_{m}){\tilde{I}}_{m}-({\gamma }_{m}+{\tilde{\alpha }}_{m}+\mu \bar{N}){\tilde{I}}_{m}.$$

The system in (4) is linear and its long-term behaviour is determined by a dominant Lyapunov exponent of the mapping. We capture the information provided by the dominant Lyapunov exponent with the pathogen-fitness function, ${W}_{\alpha }({\tilde{\alpha }}_{f},{\tilde{\alpha }}_{m},{\alpha }_{f},{\alpha }_{m})$ using techniques laid out by the ref. 55 (see also Supplemental Information). When this function is greater than 1 the mutant invades and eventually displaces^56^ the resident strain associated with the *α*_*i*_ phenotype. When the function ${W}_{\alpha }({\tilde{\alpha }}_{f},{\tilde{\alpha }}_{m},{\alpha }_{f},{\alpha }_{m})$ is less than 1 the mutant does not invade and is eliminated from the population. With these facts in mind, we say that selection acts to move *α*_*i*_ in the direction given by the sign of $\frac{\partial {W}_{\alpha }}{\partial {\tilde{\alpha }}_{i}}{\left|\right.}_{\tilde{\alpha }=\alpha }$ where $\tilde{\alpha }=\alpha$ is shorthand for ${\tilde{\alpha }}_{i}={\alpha }_{i}$ for all *i*. Specifically, when this partial derivative is positive *α*_*i*_ is increasing, and when it is negative *α*_*i*_ is decreasing.

We follow a similar procedure for the host population by introducing, into the equilibrium population, a rare mutant-type host genotype that results in a recovery rate of ${\hat{\gamma }}_{i}$ when expressed by sex-*i* hosts. We denote the numbers of susceptible and infected sex-*i* mutant-type hosts as ${\hat{S}}_{i}$ and ${\hat{I}}_{i}$, respectively. We assume that hosts are diploid, and so, strictly speaking, the hosts who contribute to ${\hat{S}}_{i}$ and ${\hat{I}}_{i}$ categories are heterozygotes (the numbers of homozygote mutants are negligible). While it remains rare, the dynamics of the mutant-host lineage can be described using

5a $$\frac{d{\hat{S}}_{f}}{dt}=	 \frac{\frac{b({\hat{\gamma }}_{f},{\gamma }_{m})}{2}({\hat{S}}_{f}+(1-v){\hat{I}}_{f})({\bar{S}}_{m}+{\bar{I}}_{m})+\frac{b({\gamma }_{f},{\hat{\gamma }}_{m})}{2}({\bar{S}}_{f}+(1-v){\bar{I}}_{f})({\hat{S}}_{m}+{\hat{I}}_{m})}{\bar{N}}\\ 	+{\hat{\gamma }}_{f}{\hat{I}}_{f}-{\hat{S}}_{f}{\beta }_{ff}{\bar{I}}_{f}-{\hat{S}}_{f}{\beta }_{fm}{\bar{I}}_{m}-\mu \bar{N}{\hat{S}}_{f}$$

5b $$\frac{d{\hat{I}}_{f}}{dt}=	 \frac{\frac{b({\hat{\gamma }}_{f},{\gamma }_{m})}{2}v{\hat{I}}_{f}({\bar{S}}_{m}+{\bar{I}}_{m})+\frac{b({\gamma }_{f},{\hat{\gamma }}_{m})}{2}v{\bar{I}}_{f}({\hat{S}}_{m}+{\hat{I}}_{m})}{\bar{N}}\\ 	+{\hat{S}}_{f}{\beta }_{ff}{\bar{I}}_{f}+{\hat{S}}_{f}{\beta }_{fm}{\bar{I}}_{m}-({\hat{\gamma }}_{f}+{\alpha }_{f}+\mu \bar{N}){\hat{I}}_{f}$$

5c $$\frac{d{\hat{S}}_{m}}{dt}=	 \frac{\frac{b({\hat{\gamma }}_{f},{\gamma }_{m})}{2}({\hat{S}}_{f}+(1-v){\hat{I}}_{f})({\bar{S}}_{m}+{\bar{I}}_{m})+\frac{b({\gamma }_{f},{\hat{\gamma }}_{m})}{2}({\bar{S}}_{f}+(1-v){\bar{I}}_{f})({\hat{S}}_{m}+{\hat{I}}_{m})}{\bar{N}}\\ 	+{\hat{\gamma }}_{m}{\hat{I}}_{m}-{\hat{S}}_{m}{\beta }_{mf}{\bar{I}}_{f}-{\hat{S}}_{m}{\beta }_{mm}{\bar{I}}_{m}-\mu \bar{N}{\hat{S}}_{m}$$

5d $$\frac{d{\hat{I}}_{m}}{dt}=	 \frac{\frac{b({\hat{\gamma }}_{f},{\gamma }_{m})}{2}v{\hat{I}}_{f}({\bar{S}}_{m}+{\bar{I}}_{m})+\frac{b({\gamma }_{f},{\hat{\gamma }}_{m})}{2}v{\bar{I}}_{f}({\hat{S}}_{m}+{\hat{I}}_{m})}{\bar{N}}\\ 	+{\hat{S}}_{m}{\beta }_{mf}{\bar{I}}_{f}+{\hat{S}}_{m}{\beta }_{mm}{\bar{I}}_{m}-({\hat{\gamma }}_{m}+{\alpha }_{m}+\mu \bar{N}){\hat{I}}_{m}.$$

The birth terms in the preceding system of equations reflect (a) the fact that the mutant host, while it is rare, mates only homozygous resident hosts and (b) only half of the matings between heterozygous mutants and homozygous residents result in mutant offspring. Since the dynamics described by (5) are linear, we can again measure fitness (this time for the host) using the dominant Lyapunov exponent. We summarise the relevant information contained in this exponent with the host fitness function ${W}_{\gamma }({\hat{\gamma }}_{f},{\hat{\gamma }}_{m},{\gamma }_{f},{\gamma }_{m})$, again using techniques outlined by ref. 55. In keeping with the description of pathogen evolution, we assert that the host’s *γ*_*i*_ is increasing when $\frac{\partial {W}_{\gamma }}{\partial {\gamma }_{i}}{\left|\right.}_{\hat{\gamma=\gamma }}$ is positive, and decreasing when this partial derivative is negative, where $\hat{\gamma }=\gamma$ is shorthand for ${\hat{\gamma }}_{i}={\gamma }_{i}$ for all *i*.

We want to identify where the action of selection takes the resident pathogen and host traits (*γ*_*i*_ and *α*_*i*_, respectively) in the long term. As mentioned above, the model is too complicated to support exact mathematical predictions. Consequently, our methods rely on numerical simulation implemented in Matlab^57^. All Matlab code is publicly available (see Code Availability).

The numerical simulation takes as its input a set of parameters and an initial estimate of the long-term result of selection on co-evolution of pathogen and host ${\alpha }_{i}^{*}$, and ${\gamma }_{i}^{*}$ for *i* = *f*, *m*. The estimate is updated by (i) finding the corresponding equilibrium solution to Equation (1) in a manner that verifies its asymptotic stability, (ii) using that equilibrium solution to estimate partial derivatives $\frac{\partial {W}_{\alpha }}{\partial {\tilde{\alpha }}_{i}}{\left|\right.}_{\tilde{\alpha=\alpha }}$ and $\frac{\partial {W}_{\gamma }}{\partial {\hat{\gamma }}_{i}}{\left|\right.}_{\hat{\gamma=\gamma }}$ for *i* = *f*, *m*, and finally (iii) incrementing or decrementing elements of the estimate following the sign of the appropriate partial derivative. Steps (i)–(iii) are repeated until the absolute value of all partial derivatives is within a tolerance of zero. The result of the simulation is an estimate of the convergence stable^58,59^ co-evolutionary outcome, assuming *α*_*f*_ and *α*_*m*_, and *γ*_*f*_ and *γ*_*m*_ can be adjusted independently. Importantly, this predicted co-evolutionary outcome also corresponds to a system in which the pathogen is established in a stable equilibrium population of hosts.

Finally, we verified numerically that the convergence-stable estimate corresponded to a two-dimensional evolutionarily stable result^60^ for pathogen and host, respectively. For this reason, we can also refer to predictions generated by our numerical simulation as a continuously stable state, in analogy to the definition established by ref. 61.

### Reporting summary

Further information on research design is available in the Nature Research Reporting Summary linked to this article.

## Supplementary information

Supplementary Information

Reporting Summary

## Data Availability

Source data are provided with this paper. The data from model simulations presented in figures are available at 10.5281/zenodo.6946414. Source data are provided with this paper.

## Source data

Source Data

## Code availability

Custom code was written using Matlab^57^ and can be found at https://github.com/evanjmitchell/on-maternity-and-immunocompetence. Code is also available at 10.5281/zenodo.6946414.

## References

[1] Abrams ET, Miller EM (2011). The roles of the immune system in women’s reproduction: evolutionary constraints and life history trade-offs. Am. J. Phys. Anthropol..

[2] Furman D (2015). Sexual dimorphism in immunity: improving our understanding of vaccine immune responses in men. Expert Rev. Vaccines.

[3] Klein SL, Flanagan KL (2016). Sex differences in immune responses. Nat. Rev. Immunol..

[4] Bupp, M. R. G., Potluri, T., Fink, A. L. & Klein, S. L. The confluence of sex hormones and aging on immunity. *Front. Immunol*. **9**, 1269 (2018). 10.3389/fimmu.2018.01269 PMC5994698 29915601

[5] Gal-Oz, S. T. et al. ImmGen report: sexual dimorphism in the immune system transcriptome. Nat. Commun. **10**, 4295 (2019). 10.1038/s41467-019-12348-6 PMC6754408 31541153

[6] Jaillon S, Berthenet K, Garlanda C (2019). Sexual dimorphism in innate immunity. Clin. Rev. Allergy Immunol..

[7] Furman D (2014). Systems analysis of sex differences reveals an immunosuppressive role for testosterone in the response to influenza vaccination. Proc. Natl Acad. Sci. USA.

[8] Jacobsen H, Klein SL (2021). Sex differences in immunity to viral infections. Front. Immunol..

[9] Ngo ST, Steyn FJ, McCombe PA (2014). Gender differences in autoimmune disease. Front. Neuroendocrinol..

[10] Keestra, S. M., Male, V. & Salali, G. D. Out of balance: the role of evolutionary mismatches in the sex disparity in autoimmune disease. *Med. Hypotheses* **151**, 110558 (2021). 10.1016/j.mehy.2021.110558 33964604

[11] Kronzer VL, Bridges SL, Davis JM (2021). Why women have more autoimmune diseases than men: an evolutionary perspective. Evolut. Appl..

[12] Clocchiatti A, Cora E, Zhang Y, Dotto GP (2016). Sexual dimorphism in cancer. Nat. Rev. Cancer.

[13] Djomkam Zune, A. L. et al. Pathogen-induced epigenetic modifications in cancers: implications for prevention, detection and treatment of cancers in Africa. *Cancers* **13**, 6051 (2021). 10.3390/cancers13236051 PMC8656768 34885162

[14] Shepherd R, Cheung AS, Pang K, Saffery R, Novakovic B (2021). Sexual dimorphism in innate immunity: the role of sex hormones and epigenetics. Front. Immunol..

[15] Shultz AJ, Sackton TB (2019). Immune genes are hotspots of shared positive selection across birds and mammals. eLife.

[16] Zuk M, Stoehr AM (2002). Immune defense and host life history. Am. Naturalist.

[17] Zuk M (2009). The sicker sex. PLoS Pathog..

[18] Stoehr AM, Kokko H (2006). Sexual dimorphism in immunocompetence: what does life-history theory predict?. Behav. Ecol..

[19] Zuk, M. & Stoehr, A. M. In *Sex Hormones and Immunity to Infection* (eds Klein, S. & Roberts, C.) Ch. 1 (Springer, 2010).

[20] Restif O, Amos W (2010). The evolution of sex-specific immune defences. Proc. R. Soc. B.

[21] Metcalf, C. J. E. & Graham, A. L. Schedule and magnitude of reproductive investment under immune trade-offs explains sex differences in immunity. *Nat. Commun*. **9**, 4391 (2018). 10.1038/s41467-018-06793-y PMC6197210 30348963

[22] Guerin LR, Prins JR, Robertson SA (2009). Regulatory t-cells and immune tolerance in pregnancy: a new target for infertility treatment?. Hum. Reprod. Update.

[23] Brazdova A, Senechal H, Peltre G, Poncet P (2016). Immune aspects of female infertility. Int. J. Fertil. Steril..

[24] Naim N, Amrit FRG, Brooke McClendon T, Yanowitz JL, Ghazi A (2020). The molecular tug of war between immunity and fertility: emergence of conserved signalling pathways and regulatory mechanisms. BioEssays.

[25] Keller CC, Eikmans M, van der Hoorn MLP, Lashley LEELO (2020). Recurrent miscarriages and the association with regulatory t cells; a systematic review. J. Reprod. Immunol..

[26] Arora N, Sadovsky Y, Dermody TS, Coyne CB (2017). Microbial vertical transmission during human pregnancy. Cell Host Microbe.

[27] van Baalen M (1998). Coevolution of recovery ability and virulence. Proc. R. Soc. B.

[28] Gipson SAY, Hall MD (2016). The evolution of sexual dimorphism and its potential impact on host-pathogen coevolution. Evolution.

[29] Lipsitch M, Siller S, Nowak M (1996). The evolution of virulence in pathogens with vertical and horizontal transmission. Evolution.

[30] Cressler CE, McLeod DV, Rozins C, van den Hoogen J, Day T (2016). The adaptive evolution of virulence: a review of theoretical predictions and empirical tests. Parasitology.

[31] Úbeda, F. & Jansen, V. The evolution of sex-specific virulence in infectious diseases. *Nat. Commun*. **7**, 13849 (2016). 10.1038/ncomms13849 PMC5159935 27959327

[32] Hawkes K, O’Connell J, Jones N, Alvarez H, Charnov E (1998). Grandmothering, menopause, and the evolution of human life histories. Proc. Natl Acad. Sci. USA.

[33] Úbeda F, Ohtsuki H, Gardner A (2014). Ecology drives intragenomic conflict over menopause. Ecol. Lett..

[34] Lahdenpera M, Gillespie DOS, Lummaa V, Russell AF (2012). Severe intergenerational reproductive conflict and the evolution of menopause. Ecol. Lett..

[35] Chapman, S. N., Lahdenperae, M., Pettay, J. E., Lynch, R. F. & Lummaa, V. Offspring fertility and grandchild survival enhanced by maternal grandmothers in a pre-industrial human society. *Sci. Rep.* **11**, 3652 (2021). 10.1038/s41598-021-83353-3 PMC7878921 33574488

[36] Day T (2002). On the evolution of virulence and the relationship between various measures of mortality. Proc. R. Soc. B.

[37] Fairweather D, Rose NR (2004). Women and autoimmune diseases. Emerg. Infect. Dis..

[38] Gleicher N, Barad DH (2007). Gender as risk factor for autoimmune diseases. J. Autoimmun..

[39] Taneja V (2021). Sexual dimorphism, aging and immunity. Vitam. Hormones.

[40] Anderson GD (2005). Sex and racial differences in pharmacological response: where is the evidence? Pharmacogenetics, pharmacokinetics, and pharmacodynamics. J. Women’s Health.

[41] Zopf Y (2008). Women encounter ADRs more often than do men. Eur. J. Clin. Pharmacol..

[42] Kim AM, Tingen CM, Woodruff TK (2010). Sex bias in trials and treatment must end. Nature.

[43] Vidaver RM, Lafleur B, Tong C, Bradshaw R, Marts SA (2000). Women subjects in NIH-funded clinical research literature: Lack of progress in both representation and analysis by sex. J. Women’s Health Gend. Based Med..

[44] Berec L (2018). Mate search and mate-finding Allee effect: on modeling mating in sex-structured population models. Theor. Ecol..

[45] Caswell H, Weeks DE (1986). Two-sex models: chaos, extinction, and other dynamic consequences of sex. Am. Naturalist.

[46] Alizon S, Michalakis Y (2015). Adaptive virulence evolution: the good old fitness-based approach. Trends Ecol. Evol..

[47] Ewald PW (1983). Host-parasite relations, vectors, and the evolution of disease severity. Annu. Rev. Ecol. Evol. Syst..

[48] Day T, Burns JG (2003). A consideration of patterns of virulence arising from host-parasite coevolution. Evolution.

[49] Beagley KW, Gockel CM (2003). Regulation of innate and adaptive immunity by the female sex hormones oestradiol and progesterone. FEMS Immunol. Med. Microbiol..

[50] Cutolo M (2004). Sex hormones influence on the immune system: basic and clinical aspects in autoimmunity. Lupus.

[51] Tan IJ, Peeva E, Zandman-Goddard G (2015). Hormonal modulation of the immune system - A spotlight on the role of progestogens. Autoimmun. Rev..

[52] Dercole, F. & Rinaldi, S. *Analysis of Evolutionary Processes: The Adaptive Dynamics Approach and Its Applications* (Princeton Univ. Press, 2008).

[53] Dieckmann U, Law R (1996). The dynamical theory of coevolution: a derivation from stochastic ecological processes. J. Math. Biol..

[54] Metz JAJ, Nisbet RM, Geritz SAH (1992). How should we define ‘fitness’ for general ecological scenarios?. Trends Ecol. Evolution.

[55] Hurford A, Cownden D, Day T (2010). Next-generation tools for evolutionary invasion analyses. J. R. Soc. Interface.

[56] Geritz SAH (2005). Resident-invader dynamics and the coexistence of similar strategies. J. Math. Biol..

[57] MATLAB. version 9.6.0 (R2019a) (The MathWorks Inc., 2019).

[58] Christiansen FB (1991). On conditions for evolutionary stability for a continuously varying character. Am. Naturalist.

[59] Leimar O (2009). Multidimensional convergence stability. Evolut. Ecol. Res..

[60] Maynard Smith, J. *Evolution and the Theory of Games* (Cambridge Univ. Press, 1982).

[61] Eshel I (1983). Evolutionary and continuous stability. J. Theor. Biol..

