# Supplementary material for: On maternity and the stronger immune response in women
Source: Nat Commun. 2022 Aug 18;13:4858. doi: 10.1038/s41467-022-32569-6 (PMC9386672; doi:10.1038/s41467-022-32569-6)
Supplement: Supplementary file 1 — Supplementary Information [file 41467_2022_32569_MOESM1_ESM.pdf]

Supplement to “On maternity and the stronger immune response in women”

Evan Mitchell[1], Andrea L. Graham[2], Francisco Úbeda[3,*†], Geoff Wild[1,†]

[1]Department of Mathematics, Western University
London, ON N6A 5B7, Canada

[2]Department of Ecology & Evolutionary Biology, Princeton University
Princeton, New Jersey 08544 USA

[3]Department of Biological Sciences, Royal Holloway, University of London
Egham, Surrey TW20 0EX, United Kingdom

Contents

1 Resident Ecological Dynamics **2**

2 Evolutionary Analysis **4**

2.1 Invasion Fitness of the Pathogen 4

2.2 Invasion Fitness of the Host 8

2.3 Numerical Determination of Joint ESSs 10

3 Results **13**

*contributed equally

†corresponding authors: f.ubeda@rhul.ac.uk, gwild@uwo.ca

3.1 No Sex-Related Differences . . . 13
3.2 Sex-Related Differences in Cost of Immune Function Only . . . 14
3.3 Sex-Related Differences in the Opportunity for Vertical Transmission Only 15
3.4 Sex-Related Differences in Cost and Vertical Transmission . . . 17

4 Extension to Immune Activation Costs Only **24**
4.1 Preamble . . . 24
4.2 Modified Model . . . 24

5 Summary of Custom Code Used in Model Analysis **33**

6 Data Availability **33**

1 Resident Ecological Dynamics

In this section, we outline the ecological model upon which our evolutionary analysis relies. As described in the main text, the host population is subdivided into four categories based on infection status and sex. We use $S_X(t)$ to denote the number of sex-X individuals ($X = f$ for female and $X = m$ for male) who, at time t, are not infected with the pathogen but are susceptible to future infection, and we use $I_X(t)$ to denote the number of sex-X individuals who are both infected and infectious at time t. We track the rate of change in the numbers of individuals in each of the four categories by modelling gains and losses through demographic processes like birth and death, and disease-related processes like transmission and recovery.

Host births occur at total rate

$$\frac{b}{2}\frac{4(S_f + I_f)(S_m + I_m)}{N} = \frac{2b(S_f + I_f)(S_m + I_m)}{N} \tag{1}$$

where $N = S_f + S_m + I_f + I_m$ is the total population size. We recognize the term $4(S_f + I_f)(S_m + I_m)/N$ as the effective size of the host population[1] (see also work by Berec[2], and Caswell & Weeks[3]). We assume the birth sex ratio to be even and so newborn individuals are equally likely to be female or male. We also assume that offspring produced by susceptible mothers are always born susceptible, but those produced by infected mothers are only born susceptible with probability $1 - v$. The remaining fraction of offspring produced by infected mothers, denoted v, are born infected. In keeping with previous work[4],

we call v the probability of vertical disease transmission.

Host death can occur for reasons related to disease status and for reasons unrelated to disease status. Death in the latter case occurs at the density-dependent per-capita rate of μN, where μ is a positive constant. Disease-related death only affects infected individuals. We use α_X to denote the additional per-capita death rate associated with infected individuals as a result of their disease status. For convenience we refer to α_X as the pathogen-induced mortality rate, and μN as the background mortality rate.

Numbers of individuals in the various categories change as their disease status changes. We model the total rate of horizontal disease transmission from sex-X individuals to sex-Y individuals ($Y = f, m$) as $S_Y \beta_{YX} I_X$. When a susceptible individual is infected we assume it automatically becomes infectious; that is to say we ignore the possibility of latent effects of the pathogen. Infected sex-X individuals recover from their infection at per-capita rate γ_X. We assume that recovered individuals are susceptible to immediate re-infection, that is to say we ignore the possibility that recovered individuals acquire even temporary immunity to the pathogen.

The models for both demographic and disease-related processes lead to the following system of differential equations:

$$\frac{dS_f}{dt} = \frac{b(S_f + (1-v)I_f)(S_m + I_m)}{N} + \gamma_f I_f - S_f \beta_{ff} I_f - S_f \beta_{fm} I_m - \mu N S_f \tag{2a}$$

$$\frac{dS_m}{dt} = \frac{b(S_f + (1-v)I_f)(S_m + I_m)}{N} + \gamma_m I_m - S_m \beta_{mf} I_f - S_m \beta_{mm} I_m - \mu N S_m \tag{2b}$$

$$\frac{dI_f}{dt} = \frac{bvI_f(S_m + I_m)}{N} + S_f \beta_{ff} I_f + S_f \beta_{fm} I_m - (\gamma_f + \alpha_f + \mu N) I_f \tag{2c}$$

$$\frac{dI_m}{dt} = \frac{bvI_f(S_m + I_m)}{N} + S_m \beta_{mf} I_f + S_m \beta_{mm} I_m - (\gamma_m + \alpha_m + \mu N) I_m. \tag{2d}$$

In the absence of the infectious disease ($I_f = I_m = 0$) the system of equations reduces to

$$\frac{dS_f}{dt} = \frac{bS_f S_m}{S_f + S_m} - \mu(S_f + S_m) S_f \tag{3a}$$

$$\frac{dS_m}{dt} = \frac{bS_f S_m}{S_f + S_m} - \mu(S_f + S_m) S_m \tag{3b}$$

The demographic rates in the reduced model are independent of sex, and so over time S_f

and S_m will tend to the same equilibrium value, call it S_0. It is straightforward to show that $S_0 = \frac{b}{4\mu}$, and so $(S_0, S_0, 0, 0)$ is the *disease-free equilibrium* solution to system (2).

It can be shown[5] that the local asymptotic stability of the disease-free equilibrium depends on the spectral radius of the positive matrix

$$\mathbf{K} = \begin{bmatrix} \frac{bv/2+S_0\beta_{ff}}{\mu(2S_0)+\alpha_f+\gamma_f} & \frac{S_0\beta_{fm}}{\mu(2S_0)+\alpha_m+\gamma_m} \\ \frac{bv/2+S_0\beta_{mf}}{\mu(2S_0)+\alpha_f+\gamma_f)} & \frac{S_0\beta_{mm}}{\mu(2S_0)+\alpha_m+\gamma_m} \end{bmatrix}. \tag{4}$$

Specifically, the disease-free equilibrium is unstable whenever the spectral radius of $\mathbf{K}$, call it R_0, is greater than unity. The disease-free equilibrium is stable when the spectral radius R_0 is less than unity.

When the disease-free equilibrium is unstable, we expect the population to tend toward an *endemic equilibrium* where positive numbers of infected individuals can be found. Though the complexity of the dynamics limits our ability to prove this claim, numerical simulation using Matlab[6] supports the idea (see for example, Figure 1). We denote the endemic equilibrium as $(\bar{S}_f, \bar{S}_m, \bar{I}_f, \bar{I}_m)$ and this equilibrium sets the stage for the invasion analyses conducted below. Importantly, as part of our invasion analyses we confirm the asymptotic stability of the endemic equilibrium numerically. Each co-evolutionary prediction we report on below (and in the main text), therefore, reflects a sequence of population states in which the pathogen endemicity persists even in the face of perturbed host numbers.

2 Evolutionary Analysis

2.1 Invasion Fitness of the Pathogen

In this subsection we develop an expression for the invasion fitness of a mutant pathogen in a system dominated by a resident pathogen strain at the endemic equilibrium. The mutant pathogen induces a per-capita mortality rate of $\tilde{\alpha}_X$ among infected sex-X individuals, which may differ from the rates induced by the resident (α_X, as mentioned above).

We assume that increased mortality is the cost a pathogen must pay in order to create new infections horizontally. Without such a cost, selection would favour unlimited growth in trasmissibility. To model the trade-off, we now insist

$$\beta_{YX} = \beta_{\max}\frac{\alpha_X}{d+\alpha_X} \quad \text{and} \quad \tilde{\beta}_{YX} = \beta_{\max}\frac{\tilde{\alpha}_X}{d+\tilde{\alpha}_X} \tag{5}$$

for common constants $\beta_{\max}, d > 0$ (Figure 2). The assumption that the transmissibility of a

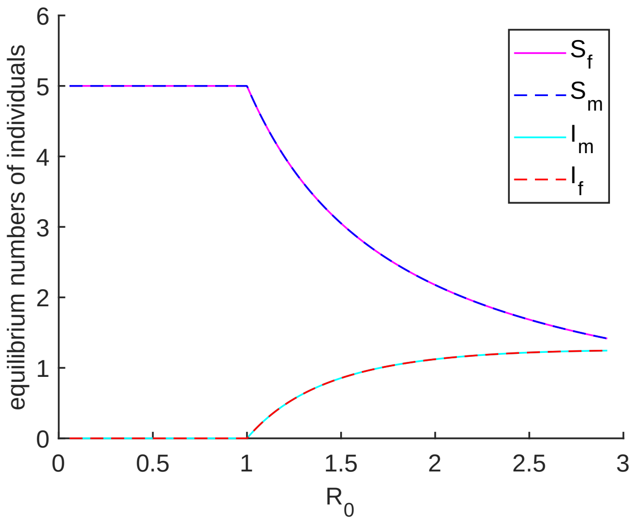

Figure 1: The relationship between long-term behaviour of solutions to system (2) and R_0, the spectral radius of the matrix $\mathbf{K}$ in equation 4. Parameters in this example $b_{\max} = 2$, $\mu = 0.1$, $v = 0.2$, $\gamma_m = \gamma_2$, $\alpha_f = \alpha_m = 1$, $\beta_{ff} = \beta_{mm} = \beta_{fm} = \beta_{mf} = 0.9$. They have been chosen so that there are no sex-specific differences in demographic patterns, and so $\bar{S}_f = \bar{S}_m$ and $\bar{I}_f = \bar{I}_m$. We see that solutions undergo a transcritical bifurcation at $R_0 = 1$, as is typically observed.

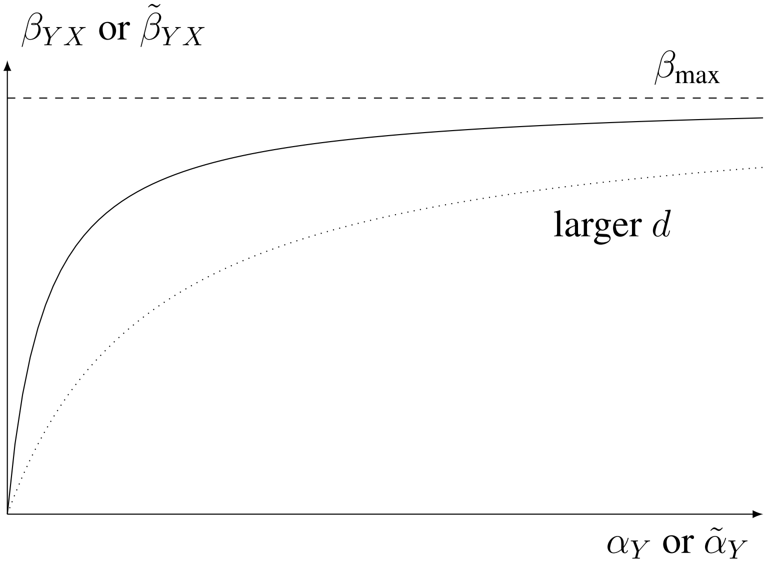

Figure 2: The relationship between transmissibility (β_{YX} or $\tilde{\beta}_{YX}$) and pathogen-induced mortality (α_Y or $\tilde{\alpha}_Y$) assumed by our model. Transmissibility saturates at a maximum value of $\beta_{\max}$ as pathogen induced mortality increases. The rate of saturation is controlled by d with larger d corresponding to slower saturation.

pathogen has virulent consequences for its host is standard, and though it is controversial, it has received empirical support[7–9].

While the mutant is rare, the growth of its subpopulation can be approximated by

$$\begin{bmatrix} \frac{d\tilde{I}_f}{dt} \\ \frac{d\tilde{I}_m}{dt} \end{bmatrix} = \mathbf{A} \begin{bmatrix} \tilde{I}_f \\ \tilde{I}_m \end{bmatrix} \tag{6a}$$

where $\tilde{I}_X(t)$ gives the number of sex-X individuals carrying the mutant pathogen at time t,

$$\mathbf{A} = \begin{bmatrix} \frac{bv(\bar{S}_m+\bar{I}_m)}{\bar{N}} + \bar{S}_f\tilde{\beta}_{ff} - (\gamma_f + \tilde{\alpha}_f + \mu\bar{N}) & \bar{S}_f\tilde{\beta}_{fm} \\ \frac{bv(\bar{S}_m+\bar{I}_m)}{\bar{N}} + \bar{S}_m\tilde{\beta}_{mf} & \bar{S}_m\tilde{\beta}_{mm} - (\gamma_m + \tilde{\alpha}_m + \mu\bar{N}) \end{bmatrix}, \tag{6b}$$

and $\bar{N} = \bar{S}_f + \bar{S}_m + \bar{I}_f + \bar{I}_m$. Rather than study the matrix in (6), we follow previous work[10] and focus on the positive matrix

$$\tilde{\mathbf{K}} = \begin{bmatrix} \frac{bv(\bar{S}_m+\bar{I}_m)/\bar{N}+\bar{S}_f\tilde{\beta}_{ff}}{\gamma_f+\tilde{\alpha}_f+\mu\bar{N}} & \frac{\bar{S}_f\tilde{\beta}_{fm}}{\gamma_m+\tilde{\alpha}_m+\mu\bar{N}} \\ \frac{bv(\bar{S}_m+\bar{I}_m)/\bar{N}+\bar{S}_m\tilde{\beta}_{mf}}{\gamma_f+\tilde{\alpha}_f+\mu\bar{N}} & \frac{\bar{S}_m\tilde{\beta}_{mm}}{\gamma_m+\tilde{\alpha}_m+\mu\bar{N}} \end{bmatrix}. \tag{7}$$

The spectral radius of $\tilde{\mathbf{K}}$ gives the invasion fitness of the rare mutant pathogen, W_p. If $W_p > 1$ the number of infections involving the mutant is growing, whereas if $W_p < 1$ that number is declining. In the former case, the mutant will successfully invade and, under a reasonable set of conditions[11], take the place of the resident. In the latter case, the mutant is eliminated from the population.

The direction of evolution of α_X favoured by selection at a particular instant in time is given by the sign of the partial derivative $\partial_{\tilde{\alpha}_X} W_p$ evaluated when $\tilde{\alpha}_f = \alpha_f$ and $\tilde{\alpha}_m = \alpha_m$. Directional selection can drive the parasite toward an evolutionarily singular state, for now denoted $\alpha_f = \alpha_f^*$ and $\alpha_m = \alpha_m^*$, where directional selective forces vanish. The parasite fitness W_p should be (at least) maximized in the variables $\tilde{\alpha}_f$ and $\tilde{\alpha}_m$ in a neighbourhood of the evolutionarily singular state, if it is to be considered an evolutionarily stable strategy (ESS). The local evolutionary stability of an evolutionarily singular state is guaranteed when the matrix of second partial derivatives of W_p is negative definite.

We evaluate W_p numerically using a custom Matlab[6] function called `Wp.m` (available at https://github.com/evanjmitchell/on-maternity-and-immunocompetence). We can use this function to arrive at finite-difference approximations for the various partial derivatives needed to determine the direction of evolution favoured by selection, and the evolutionary stability of singular states. First-order derivatives are estimated using the centred finite difference; for example,

$$\partial_{\tilde{\alpha}_f} W_p \approx \frac{W_p(\alpha_f + \delta, \alpha_m) - W_p(\alpha_f - \delta, \alpha_m)}{2\delta} \tag{8a}$$

where it is understood that $\delta > 0$ is small, and that W_p depends on many other inputs. Second-order derivatives are also estimated using a centred finite-difference approximation; for example,

$$\begin{aligned} \partial^2_{\tilde{\alpha}_f} W_p &\approx \frac{W_p(\alpha_f + \delta, \alpha_m) - 2 + W_p(\alpha_f - \delta, \alpha_m)}{\delta^2} \\ \partial_{\tilde{\alpha}_f \tilde{\alpha}_m} W_p &\approx \frac{W_p(\alpha_f + \delta, \alpha_m + \delta) - W_p(\alpha_f + \delta, \alpha_m) - W_p(\alpha_f, \alpha_m + \delta)}{2\delta^2} + \frac{2}{2\delta^2} \\ &\quad + \frac{W_p(\alpha_f - \delta, \alpha_m - \delta) - W_p(\alpha_f - \delta, \alpha_m) - W_p(\alpha_f, \alpha_m - \delta)}{2\delta^2} \end{aligned} \tag{8b}$$

where it is understood that $W_p = 1$ whenever $\tilde{\alpha}_X = \alpha_X$ for $X = f, m$.

2.2 *Invasion Fitness of the Host*

We now turn our attention to a rare mutant strain of host in a system dominated by the resident host strain at the endemic equilibrium. The mutant sex-X host recovers from infections at rate $\hat{\gamma}_X$ which may differ from the recovery rate of the resident (γ_X as mentioned above).

The cost of a speedier recovery in our model is reduced fecundity [12,13]. Specifically, we model resident birth rate b as

$$b(\gamma_f, \gamma_m) = b_{\text{max}}\, e^{-c_f \gamma_f^2}\, e^{-c_m \gamma_m^2} \tag{9a}$$

where the Gaussian terms capture the reduction in maximal fecundity b_{max} that comes with increased immune activity for males and females, respectively. The parameter $c_X > 0$ reflects the sex-specific cost of immune function, with larger c_X resulting in a sharper drop in reproductive output (Figure 3). Mutant birth rates are modelled in a similar manner with

$$b(\hat{\gamma}_f, \gamma_m) = b_{\text{max}}\, e^{-c_f \hat{\gamma}_f^2}\, e^{-c_m \gamma_m^2} \tag{9b}$$

describing the rate at which mutant females produce offspring, and

$$b(\gamma_f, \hat{\gamma}_m) = b_{\text{max}}\, e^{-c_f \gamma_f^2}\, e^{-c_m \hat{\gamma}_m^2} \tag{9c}$$

doing the same for mutant males.

Let $\hat{S}_X(t)$ denote the number of mutant hosts that are not infected at time t, and let $\hat{I}_X(t)$ denote the number of mutant hosts that are infected (with the resident pathogen strain) at time t. Then, the growth of the rare mutant host can be approximated using

$$\begin{bmatrix} \frac{d\hat{S}_f}{dt} \\ \frac{d\hat{S}_m}{dt} \\ \frac{d\hat{I}_f}{dt} \\ \frac{d\hat{I}_m}{dt} \end{bmatrix} = (\mathbf{F} - \mathbf{V}) \begin{bmatrix} \hat{S}_f \\ \hat{S}_m \\ \hat{I}_f \\ \hat{I}_m \end{bmatrix} \tag{10a}$$

where

$$\mathbf{F} = \frac{1}{2} \begin{bmatrix} \frac{b(\hat{\gamma}_f,\gamma_m)(\bar{S}_m+\bar{I}_m)}{\bar{N}} & \frac{b(\gamma_f,\hat{\gamma}_m)(\bar{S}_f+(1-v)\bar{I}_f)}{\bar{N}} & \frac{(1-v)b(\hat{\gamma}_f,\gamma_m)(\bar{S}_m+\bar{I}_m)}{\bar{N}} & \frac{b(\gamma_f,\hat{\gamma}_m)(\bar{S}_f+(1-v)\bar{I}_f)}{\bar{N}} \\ \frac{b(\hat{\gamma}_f,\gamma_m)(\bar{S}_m+\bar{I}_m)}{\bar{N}} & \frac{b(\gamma_f,\hat{\gamma}_m)(\bar{S}_f+(1-v)\bar{I}_f)}{\bar{N}} & \frac{(1-v)b(\hat{\gamma}_f,\gamma_m)(\bar{S}_m+\bar{I}_m)}{\bar{N}} & \frac{b(\gamma_f,\hat{\gamma}_m)(\bar{S}_f+(1-v)\bar{I}_f)}{\bar{N}} \\ 0 & \frac{b(\gamma_f,\hat{\gamma}_m)v\bar{I}_f}{\bar{N}} & \frac{vb(\hat{\gamma}_f,\gamma_m)(\bar{S}_m+\bar{I}_m)}{\bar{N}} & \frac{b(\gamma_f,\hat{\gamma}_m)v\bar{I}_f}{\bar{N}} \\ 0 & \frac{b(\gamma_f,\hat{\gamma}_m)v\bar{I}_f}{\bar{N}} & \frac{vb(\hat{\gamma}_f,\gamma_m)(\bar{S}_m+\bar{I}_m)}{\bar{N}} & \frac{b(\gamma_f,\hat{\gamma}_m)v\bar{I}_f}{\bar{N}} \end{bmatrix} \tag{10b}$$

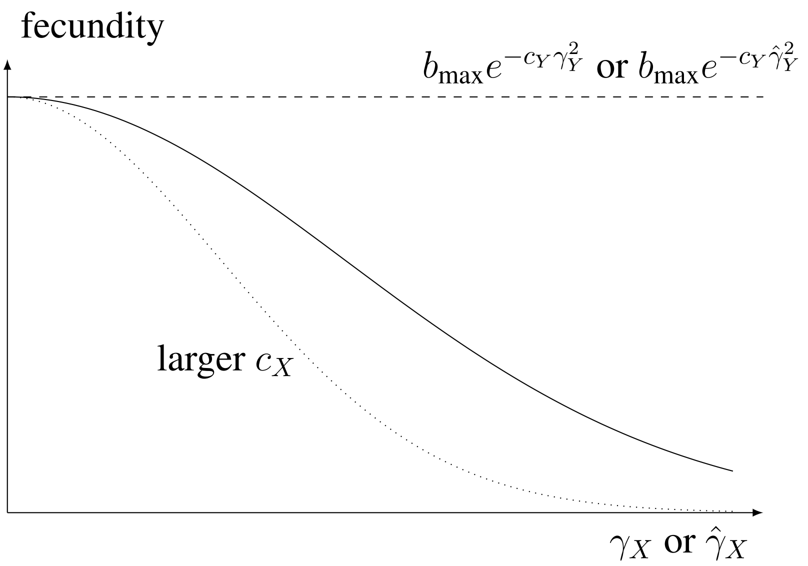

Figure 3: The relationship between host fecundity and recovery rate (γ_X or $\hat{\gamma}_X$) as described by equation (9). Fecundity falls off in a Gaussian fashion as recovery rate increases, with a larger value of c_X corresponding to a more rapid decline. Maximal fecundity depends on the parameter $b_{\max}$ as well as the recovery rate of mates, captured by $e^{-c_Y\gamma_Y^2}$ or $e^{-c_Y\hat{\gamma}_Y^2}$.

stores information about birth rates (fecundity) and

$$\mathbf{V} = \begin{bmatrix} \beta_{ff}\bar{I}_f + \beta_{fm}\bar{I}_m + \mu\bar{N} & 0 & -\hat{\gamma}_f & 0 \\ 0 & \beta_{mf}\bar{I}_f + \beta_{mm}\bar{I}_m + \mu\bar{N} & 0 & -\hat{\gamma}_m \\ -\beta_{ff}\bar{I}_f - \beta_{fm}\bar{I}_m & 0 & \hat{\gamma}_f + \alpha_f + \mu\bar{N} & 0 \\ 0 & -\beta_{mf}\bar{I}_f - \beta_{mm}\bar{I}_m & 0 & \hat{\gamma}_m + \alpha_m + \mu\bar{N} \end{bmatrix} \tag{10c}$$

stores information about loss rates from the different categories of mutant individuals. The factor of $1/2$ that appears in the matrix $\mathbf{F}$ reflects the fact that we are assuming the host is diploid, with mutant hosts being heterozygotes at a particular locus and residents being homozygotes at the same locus. For its part, the matrix $\mathbf{V}$ generates the stochastic process that would result from tracking a focal member of the mutant-host lineage as it transitions among different states over the course of its life. This generator matrix is invertible and its inverse, denoted $\mathbf{V}^{-1}$, is given by

$$\begin{bmatrix} (\mu\bar{N} + \alpha_f + \hat{\gamma}_f)\tau_f & 0 & \hat{\gamma}_f\tau_f & 0 \\ 0 & (\mu\bar{N} + \alpha_m + \hat{\gamma}_m)\tau_m & 0 & \hat{\gamma}_m\tau_m \\ (\beta_{ff}\bar{I}_f + \beta_{fm}\bar{I}_m)\tau_f & 0 & (\mu\bar{N} + \beta_{ff}\bar{I}_f + \beta_{fm}\bar{I}_m)\tau_f & 0 \\ 0 & (\beta_{mf}\bar{I}_f + \beta_{mm}\bar{I}_m)\tau_m & 0 & (\mu\bar{N} + \beta_{mf}\bar{I}_f + \beta_{mm}\bar{I}_m)\tau_m \end{bmatrix} \tag{11}$$

where

$$\tau_f = \frac{1}{\mu\bar{N}(\mu\bar{N} + \alpha_f + \hat{\gamma}_f) + (\mu\bar{N} + \alpha_f)(\beta_{ff}\bar{I}_f + \beta_{fm}\bar{I}_m)}$$

$$= \frac{1}{\mu\bar{N}\hat{\gamma}_f + (\mu\bar{N} + \alpha_f)(\mu\bar{N} + \beta_{ff}\bar{I}_f + \beta_{fm}\bar{I}_m)} \quad (12)$$

and

$$\tau_m = \frac{1}{\mu\bar{N}(\mu\bar{N} + \alpha_m + \hat{\gamma}_m) + (\mu\bar{N} + \alpha_m)(\beta_{mf}\bar{I}_f + \beta_{mm}\bar{I}_m)}$$

$$= \frac{1}{\mu\bar{N}\hat{\gamma}_m + (\mu\bar{N} + \alpha_m)(\mu\bar{N} + \beta_{mf}\bar{I}_f + \beta_{mm}\bar{I}_m)}. \quad (13)$$

The entry found in row i and column j of the matrix $\mathbf{V}^{-1}$ gives the average time an individual currently found in category j expects to spends in category i, going forward.

Rather than using $\mathbf{F} - \mathbf{V}$ to determine host fitness, we again follow previous authors[10] and work with the matrix,

$$\hat{\mathbf{K}} = \mathbf{F}\mathbf{V}^{-1}. \quad (14)$$

The spectral radius of $\hat{\mathbf{K}}$ gives host fitness, W_h. As the notation suggests, $\hat{\mathbf{K}}$ is the host analogue to the matrix $\tilde{\mathbf{K}}$ developed for the pathogen in equation (7).

In keeping with our discussion of pathogen fitness, $W_h > 1$ implies that the mutant lineage of host is proliferating and will eventually displace the incumbent. By contrast, $W_h < 1$ implies the lineage is declining toward extinction. Still in keeping with the previous discussion, we use the sign of $\partial_{\hat{\gamma}_X} W_h$ – evaluated when $\hat{\gamma}_X = \gamma_X$, for $X = f, m$ – to determine the direction in which selection moves γ_X at any point in time. We also continue to use second partial derivatives $\partial_{\hat{\gamma}_X\hat{\gamma}_Y} W_h$ – evaluated at those singular strategies where first-partial derivatives vanish – to assess evolutionary stability.

A custom Matlab function – titled `Wh.m` and available at https://github.com/evanjmitchell/on-maternity-and-immunocompetence – is used to calculate host invasion fitness. It also serves as the computational basis for finite-difference approximation of partial derivatives as outlined in equations (8), mutatis mutandis.

2.3 Numerical Determination of Joint ESSs

Given the complexity of the model, we must estimate the result of selection-driven coevolution numerically. We start by finding evolutionarily singular quadruples α_f^*, α_m^*, γ_f^*,

γ_m^* for a given set of model parameters. Briefly, evolutionarily singular quadruples are found by guessing their identity, then updating that guess iteratively by moving in the direction of the co-evolutionary selection gradient $(\partial_{\tilde{\alpha}_f} W_p,\ \partial_{\tilde{\alpha}_m} W_p,\ \partial_{\hat{\gamma}_f} W_h,\ \partial_{\hat{\gamma}_m} W_h)$ (where it is understood that partial derivatives are evaluated at the point in trait space corresponding to the current guess). We stop updating when the magnitude of the selection gradient falls below some small threshold value, or (as a fail-safe) when we exceed some maximum number of iterations.

The method we used to estimate evolutionarily singular quadruples is presented as pseudocode in Algorithm 1. The method itself prompts us to describe the quadruple as being evolutionarily attainable in the sense that it satisfies a multi-dimensional generalization of the convergence-stability concept[14,15]. To assess the evolutionary stability of the singular strategy, we use finite-difference approximations of second partial derivatives of W_p and W_h, respectively, to create separate 2×2 Hessian matrices (one for the pathogen, and a second for the host). The eigenvalues of the Hessian matrices are guaranteed to be real, and if both Hessians have only negative eigenvalues when evaluated at the singular point, we classify the corresponding singular quadruple as a joint ESS.

We implemented our numerical method using Matlab[6]. The central script used is reflected in the demo, `maindemo.m` (`https://github.com/evanjmitchell/on-maternity-and-immunocompetence`). The demo relies on other custom functions we have created, and so readers interested in running it should consult Table 2 at the end of this supplement.

The demo itself can be run on a standard laptop in about 0.5 seconds. Its output is written to a comma-delimited file that contains information on parameter values used, estimates obtained, and whether those estimates are evolutionarily stable. The output file also contains information about the equilibrium state of the population at the joint ESS, the magnitude of the selection gradient at the joint ESS (it should be close to zero but not necessarily equal to zero), and whether the fail-safe maximum number of iterations (set to 100 000) was exceeded. For further analysis, output can be read back into Matlab as a table, or read into Python (using the pandas module) or R as a dataframe.

Algorithm 1 Computational method by which an evolutionarily singular quadruple of traits α_f^*, α_m^*, γ_f^*, γ_m^* comes to be estimated. The steps involved also allow us to categorize the quadruple as convergence stable.

$\alpha_f^* \leftarrow$ random guess
$\alpha_m^* \leftarrow$ random guess
$\gamma_f^* \leftarrow$ random guess
$\gamma_m^* \leftarrow$ random guess
Grad $\leftarrow (1,\, 1,\, 1,\, 1)$
while $\|$Grad$\|$ $>$ small positive tolerance **do**
  Use α_f^*, α_m^*, γ_f^*, γ_m^* and supplied parameters to find resident endemic equilibrium iteratively, thus confirming its position and its asymptotic stability.
  Use endemic equilibrium and parameters to determine invasion fitness of pathogen mutants with traits near α_f^*, α_m^*.
  Use endemic equilibrium and parameters to determine invasion fitness of host mutants with traits near γ_f^*, γ_m^*.
  Use pathogen invasion fitness to establish finite-difference approximations of $\partial_{\tilde{\alpha}_f} W_p$ and $\partial_{\tilde{\alpha}_m} W_p$ evaluated at the system's current position in trait space.
  Use host invasion fitness to establish finite-difference approximations of $\partial_{\hat{\gamma}_f} W_h$ and $\partial_{\hat{\alpha}_m} W_h$ evaluated at the system's current position in trait space.
  Grad $\leftarrow (\partial_{\tilde{\alpha}_f} W_p,\, \partial_{\tilde{\alpha}_m} W_p,\, \partial_{\hat{\gamma}_f} W_h,\, \partial_{\hat{\gamma}_m} W_h)$
  $\alpha_f^* \leftarrow \alpha_f^* +$ mutational step $\cdot\, \partial_{\tilde{\alpha}_f} W_p$
  $\alpha_m^* \leftarrow \alpha_m^* +$ mutational step $\cdot\, \partial_{\tilde{\alpha}_m} W_p$
  $\gamma_f^* \leftarrow \gamma_f^* +$ mutational step $\cdot\, \partial_{\hat{\gamma}_f} W_h$
  $\gamma_m^* \leftarrow \gamma_m^* +$ mutational step $\cdot\, \partial_{\hat{\gamma}_m} W_h$
end while
$/*$ At this point the method returns an estimate of a convergence-stable quadruple. $*/$

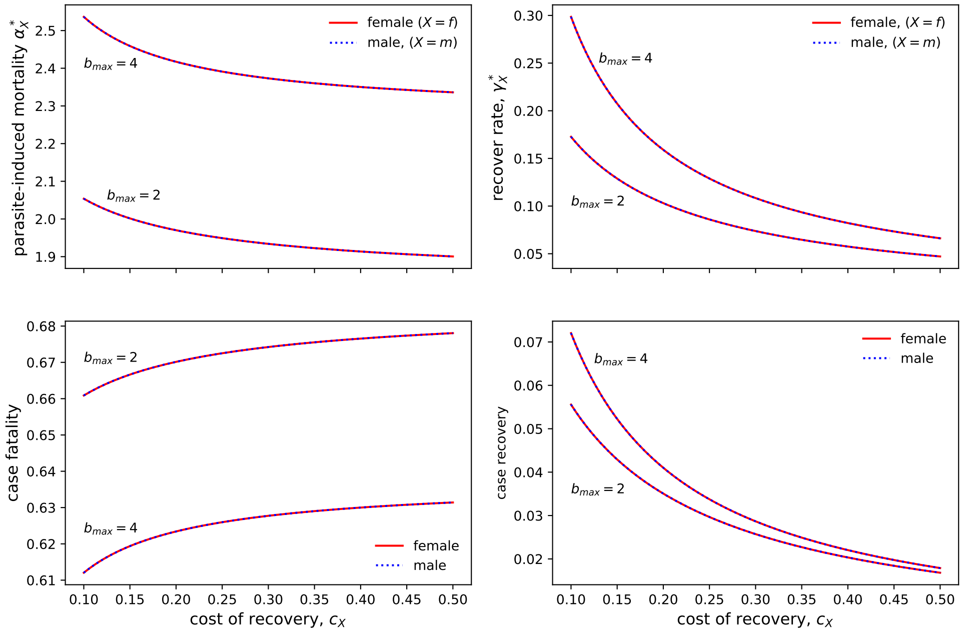

Figure 4: Predictions arising from a model with no sex-related differences; specifically $v = 0$ and $c_f = c_m = c_X$. As expected, selection in this case leads to $\alpha_f^* = \alpha_m^*$ and $\gamma_f^* = \gamma_m^*$. Additional parameters include $\mu = 0.1$, $\beta_{\max} = 1.1$, and $d = 4$ (for all X, Y).

3 Results

3.1 No Sex-Related Differences

When both $c_f = c_m$ and $v = 0$ there are no sex-related differences in the model, save the fact that pathogen-induced mortality α_X and recovery γ_X, thought of as traits, can be expressed in a way that depends on the sex of the host. Unsurprisingly, when both $c_f = c_m$ and $v = 0$ the model predicts that selection leads to a situation in which $\alpha_f^* = \alpha_m^*$ and $\gamma_f^* = \gamma_m^*$ (e.g. Figure 4).

What is perhaps surprising is the predictions related to the *case mortality rate* (sometimes *case fatality*), defined as the probability of an infected individual dying because of its infection. We see that this often-used measure of virulence[16] actually increases as the ESS rate of pathogen-induced mortality declines. Equally surprising might be the fact that the *case recovery rate*, defined as the probability an infected individual recovers from its infec-

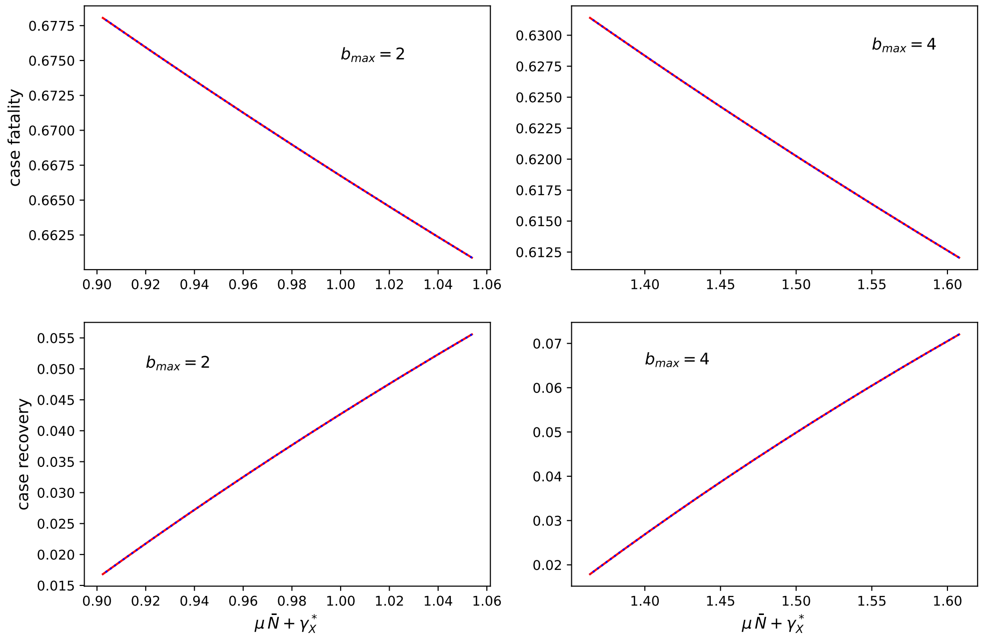

Figure 5: Predictions from Figure 4 re-plotted to show the relationship between case fatality and case recovery, respectively, and $\mu \bar{N} + \gamma_X^*$ the rate at which infections end for reasons other than pathogen-induced death. Qualitatively similar results appear in Day & Burns[17] (their Figure 2).

tion, declines with the ESS rate of pathogen-induced mortality. The paradoxical nature of these predictions is resolved when one recognizes that the effect a pathogen has on its host is also affected by host immune function[17], captured here by γ_X^*. In fact, numerical predictions made by our model suggest patterns that are qualitatively similar to those presented in other theoretical studies of host-pathogen co-evolution (compare Figure 5 to Figure 2 in work by Day & Burns[17])

3.2 Sex-Related Differences in Cost of Immune Function Only

When the only difference between the sexes stems from the cost they pay to maintain their immune systems, we find pathogen-induced mortality rate and host recovery rate evolve to different levels in the different sexes. Model predictions for this case are presented in Figure 6. We find that the ESS recovery rate γ_X^* is greater in the sex that pays the lower cost c_X for immune function. We also find that the ESS level of pathogen-induced mortality α_X^* is

greater in the sex that pays the lower cost c_X for immune function. Evidently, a cheaper immune system allows the host to mount a more aggressive defence which, in turn, elicits a more aggressive response from the pathogen. This explanation is reinforced by the fact that the most aggressive ESS outcomes presented in Figure 6 correspond to scenarios in which the average cost of immune function is lowest (average cost $= 0.1$).

Figure 6 shows, once again, that lower ESS levels of pathogen-induced mortality correspond to a reduction in the case fatality rate and to an increase in the case recovery rate. The apparent paradoxic can, once again, be resolved by recognizing that there is a co-evolutionary process at work. As a result, changes in α_X^* occur alongside changes in γ_X^* and even $\mu\bar{N}$, and each of these quantities factors into things like the case fatality and case recovery rates.

Figure 6 shows that, when immune function is cheaper for females, host-pathogen co-evolution leads to greater recovery rates in females (γ_f^* and case recovery) and higher case fatality rates in males. It should be added, though, that reduced immune cost for females does lead to ESS parasite-induced mortality rates that satisfy $\alpha_m^* < \alpha_f^*$.

3.3 Sex-Related Differences in the Opportunity for Vertical Transmission Only

When the only difference between the sexes is due to only females allowing pathogens to transmit vertically, we again find pathogen-induced mortality rate and host recovery rate evolve to different levels in the different sexes (Figure 7).

For females, we find that increasing the likelihood of vertical transmission leads to lower ESS rates of pathogen-induced mortality α_f^* and increased ESS recovery rates γ_f^*. This pattern is qualitatively opposite to the one identified in the previous subsection, where α_X^* and γ_X^* varied together. Now, we see (for females at least) that α_X^* and γ_X^* move in opposite directions. Vertical transmission offers pathogens an incentive to extend the duration of infection, and reduced α_f^* is the result. Vertical transmission also provides an incentive for increased γ_f^* as vertical transmission, itself, implies that infected females suffer the cost of producing lower-quality infected offspring. Ultimately, though, the possibility of vertical transmission reduces the case fatality rate and increases the case recovery rate for females.

For males, increased rate of vertical transmission weakly increases the rate of pathogen-induced mortality α_m^* and increases the rate of recovery γ_m^*. The net effect of the co-evolutionary process, though, does not noticeably change the case fatality rate for males, at least for the parameters investigated in Figure 7. Slight increases in case recovery rates

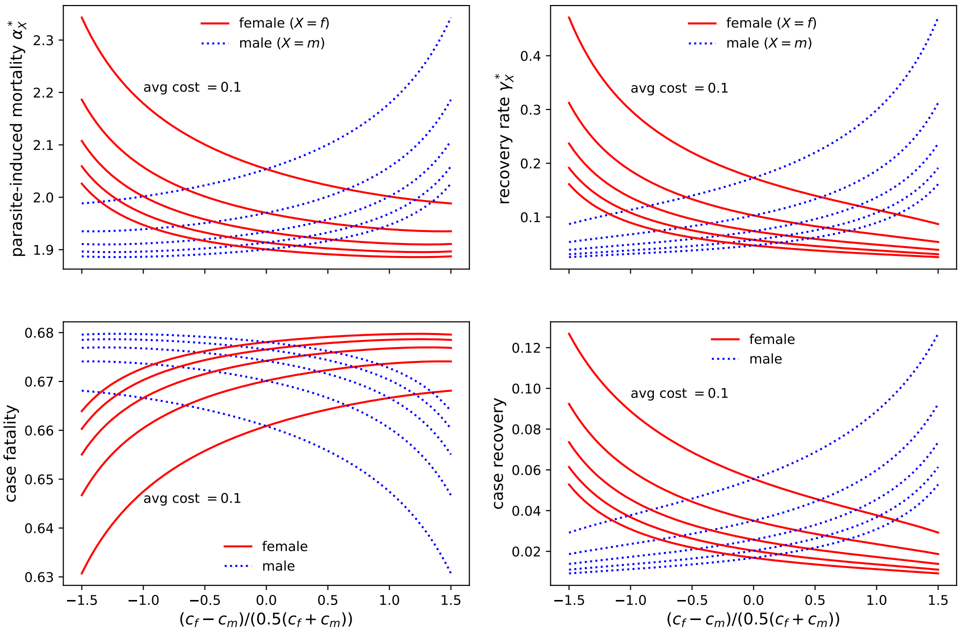

Figure 6: Predictions arising from a model with no vertical disease transmission ($v = 0$). Relative difference in cost of host immune function, measured as $(c_f - c_m)/(0.5(c_f + c_m))$ changes along the horizontal axis. Curves for different average cost $0.5(c_f + c_m)$ are presented and vary continuously from 0.1 (marked) to 0.5 in steps of size 0.1. Additional parameters include $b_{\text{max}} = 2$, $\mu = 0.1$, $\beta_{\text{max}} = 1.1$, and $d = 4$ (for all X, Y). Predictions for females can be refelcted in the vertical line through the origin to obtain predictions for males.

with increased likelihood of vertical transmission are also evident (Figure 7).

It is worth noting that an erratic pattern can be seen for some curves presented in Figure 7 when vertical transmission rates are high. These erratic patterns are numerical artefacts resulting from our method's inability to resolve ESS trait levels when one or more ESS value is near enough to zero. In the case of Figure 7, the offending trait is α_f^* when vertical transmission and the cost of immune function are both high(er).

3.4 Sex-Related Differences in Cost and Vertical Transmission

When we simultaneously allow vertical transmission and sex-specific cost of immune function, a range of co-evolutionary outcomes occurs (e.g. Figure 8). Particular outcomes, though, catch our attention. Those noteworthy outcomes are ones in which evolved virulence (defined either as α_X^* or as case mortality rate) is greater for males, yet evolved recovery (defined either as γ_X^* or the probability of recovery) is greater for females. Such outcomes are seen most readily – but not exclusively – when cost of immune function is greater in males and vertical transmission rates are sufficiently high.

The observation that males evolve to recover less readily than females, yet suffer the greater evolved mortality risk of infection is surprising at first glance. The result is driven by the fact that negative consequences of infection are also mediated by vertical transmission. As mentioned above, vertical transmission introduces an extra cost for infected females. Specifically, vertical transmission implies that infected females will lose fitness because they produce lower-quality offspring on average. This extra cost can be mitigated by females who ramp up their immune function (relative to, say, situations in which $v = 0$). Males, by comparison, have no similar means of adjusting their exposure to the risk associated with the production of low-quality infected offspring, and so the pattern we observe emerges.

Our model predicts that the scope for greater co-evolved virulence in males and greater co-evolved recovery in females is reduced as (i) maximum disease transmissibility, β_{max}, rises (Figure 9), and (ii) disease transmissibility saturates more quickly, i.e. as d decreases (Figure 10). By contrast, the scope for greater co-evolved virulence in males and greater co-evolved recovery in females decreases as (iii) host immunity becomes cheaper (Figure 11), and (iv) the maximum fecundity of the host – the size of the reserve from which the host pays for its immune response – increases (Figure 12). Overall, the effects (i)-(iv) work by bringing the perspectives of the sexes into closer alignment, either because the

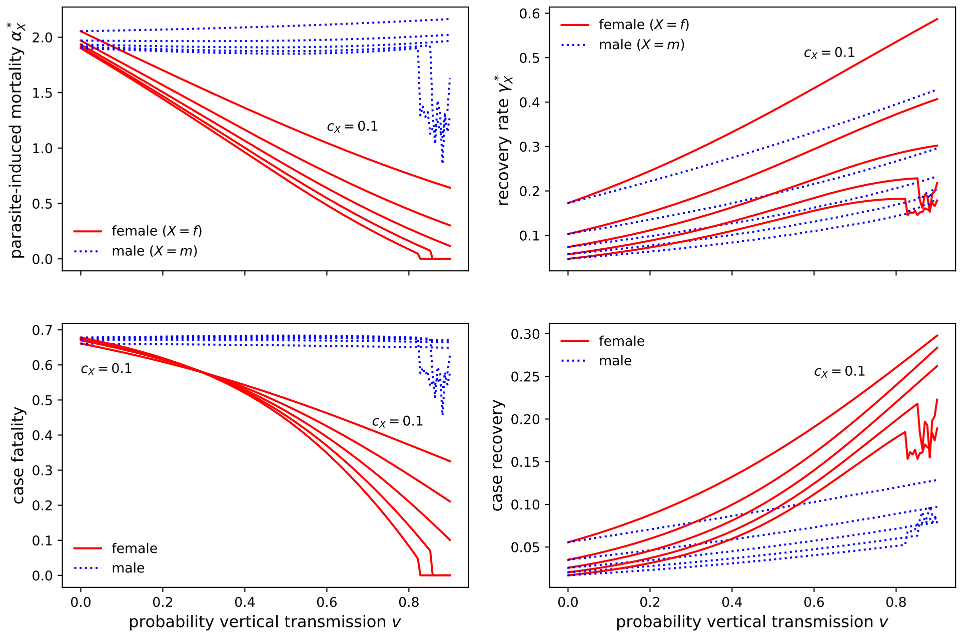

Figure 7: Predictions arising from a model with no sex difference in cost of immune function. Probability of vertical transmission, v, changes along the horizontal axis. Curves for different cost values $c_f = c_m = c_X$ are presented and vary continuously from 0.1 (marked) to 0.5 in steps of size 0.1. Female (red) and male (blue) curves meet at $v = 0$. Note that the relative order of female (red) case-fatality curves in the bottom left panel becomes reversed as one moves left to right. Additional parameters include $b_{\text{max}} = 2$, $\mu = 0.1$, $\beta_{\text{max}} = 1.1$, and $d = 4$ (for all X, Y). Erratic portions of curve reflect the numerical procedure's (expected) inability to resolve ESS values when one character value (in this case α_f^*) becomes small.

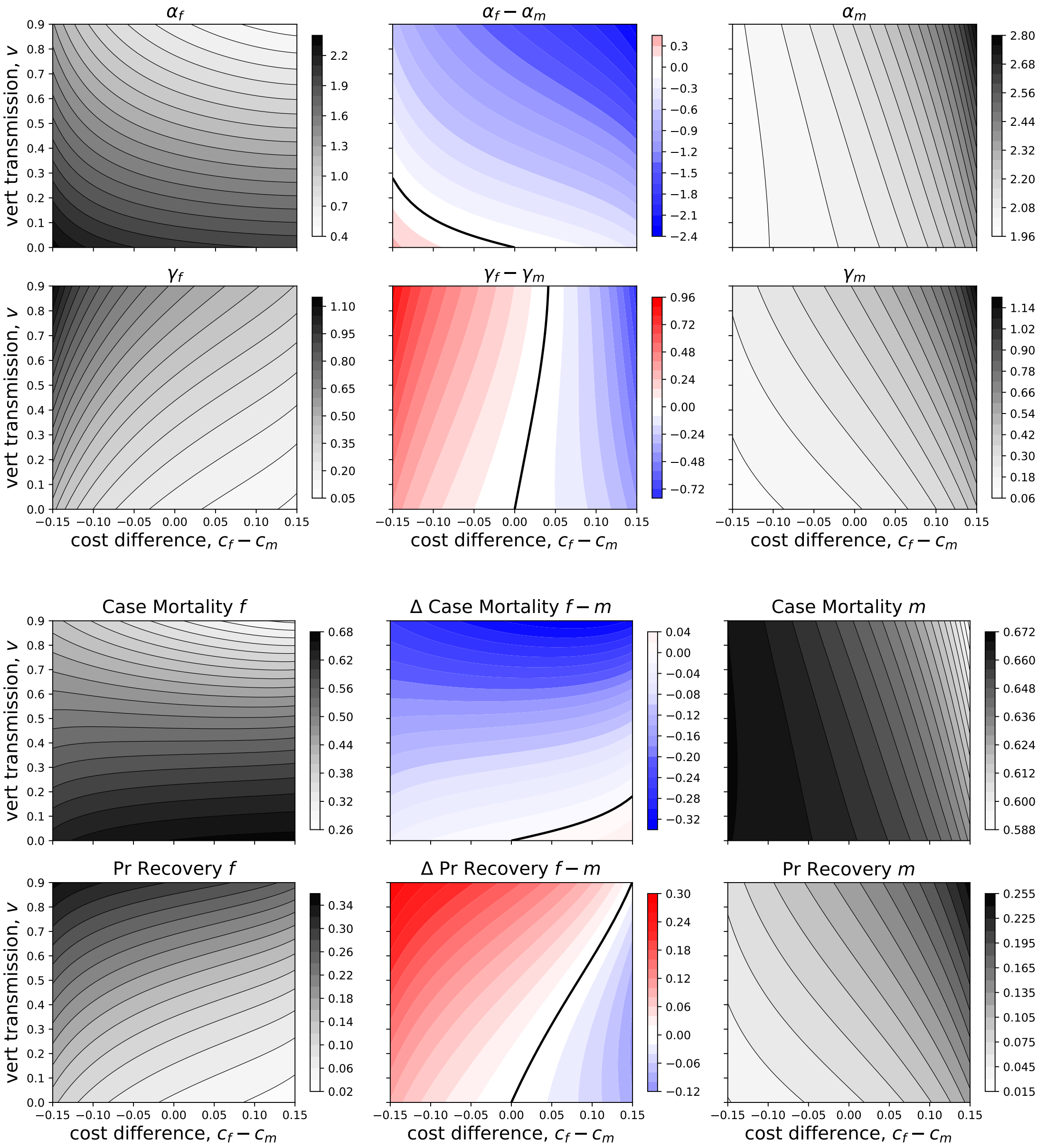

Figure 8: Changes in ESS predictions as vertical transmission and difference in the cost of immune function changes. Changes in case fatality rate and case recovery rates are also presented. Center panels allow quick comparison of outcomes for the sexes, with red areas corresponding to greater values in females and blue areas corresponding to greater values in males. Results are based on $b_{max} = 2$, $\mu = 0.1$, $0.5(c_f + c_m) = 0.1$, $\beta_{\max} = 1.1$, and $d = 4$ (for all X, Y). Black curves in centre panels correspond to zero differences between the sexes and separate red regions from blue ones.

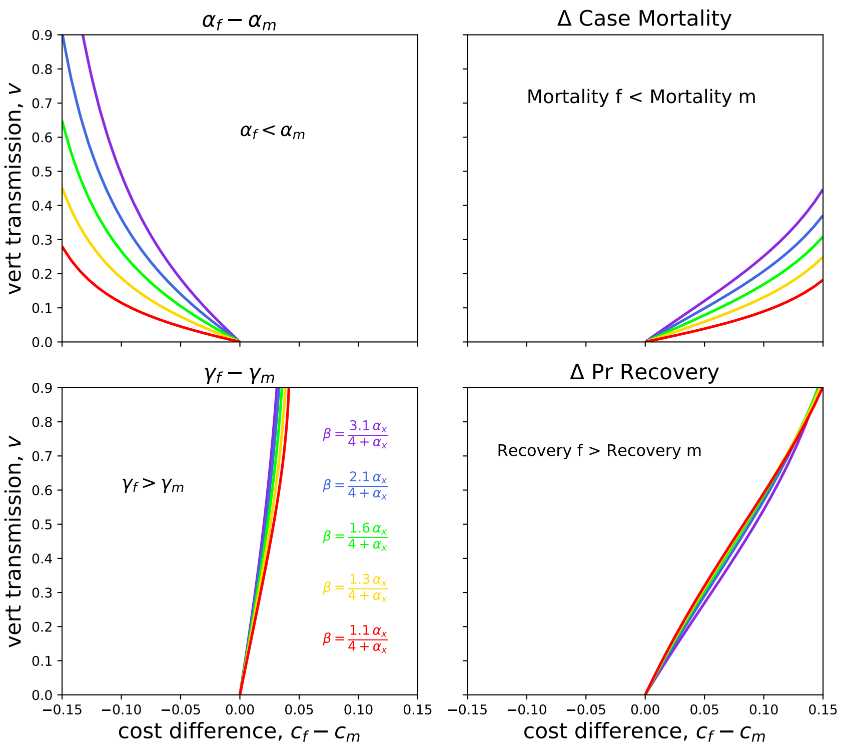

Figure 9: The area encompassed by regions in which (i) virulence (defined as either disease-related mortality or case mortality) is greater in males *and* (ii) recovery (defined as either recovery rate or probability of recovery) is greater in females shrinks when, all else being equal, maximum transmissibility $\beta_{\max}$ increases (red to violet curves). Results are based on $b_{max} = 2$, $\mu = 0.1$, $0.5(c_f + c_m) = 0.1$.

cost disparities are lessened directly, or because the consequences of producing infected offspring become less dire.

The pattern described above – greater co-evolved virulence in males and greater co-evolved recovery in females is reduced – is not a quirk of the particular additional parameters with which we supplied our numerical procedure. In fact, we investigated numerous other parameter sets and have found appreciable fractions of the space illustrated in Figure 8 correspond to the same noteworthy qualitative pattern (Table 1). It should be emphasized that the parameter sets presented in Table 1 were ones that allowed us to resolve ESS trait values numerically over the area spanned by the axes in Figure 8. Parameters were otherwise chosen arbitrarily, and so Table 1 does not reflect a systematic exploration of parameter space. Nevertheless, Table 1 suggest to us that the idea that strong recovery by females in

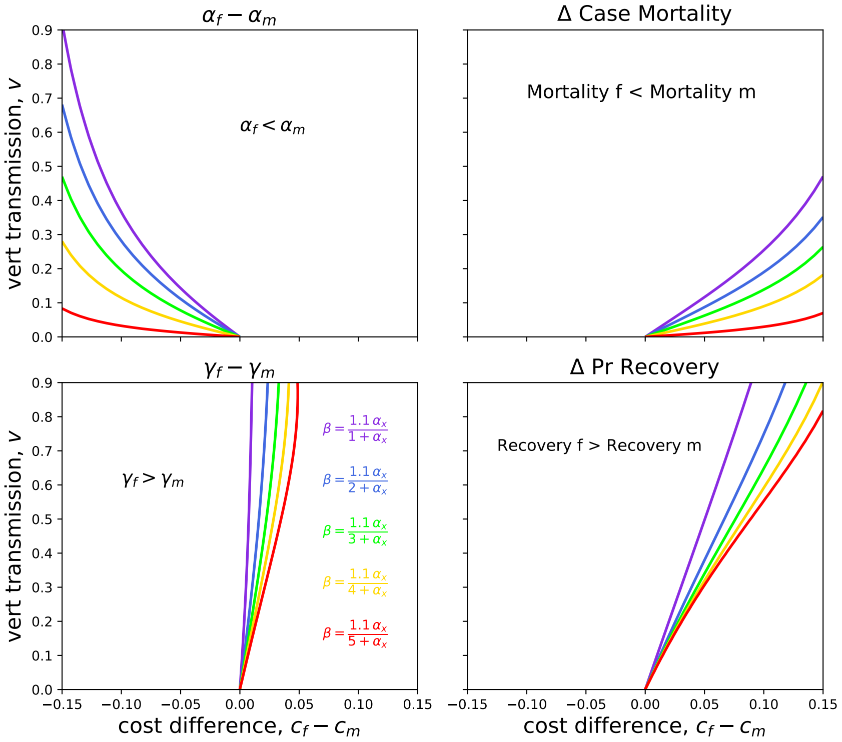

Figure 10: The area encompassed by regions in which (i) virulence (defined as either disease-related mortality or case mortality) is greater in males *and* (ii) recovery (defined as either recovery rate or probability of recovery) is greater in females shrinks when, all else being equal, transmissibility shifts from low saturation (large d, red curves) to high saturation (small d, violet curves). Results are based on $b_{max} = 2$, $\mu = 0.1$, $0.5(c_f + c_m) = 0.1$.

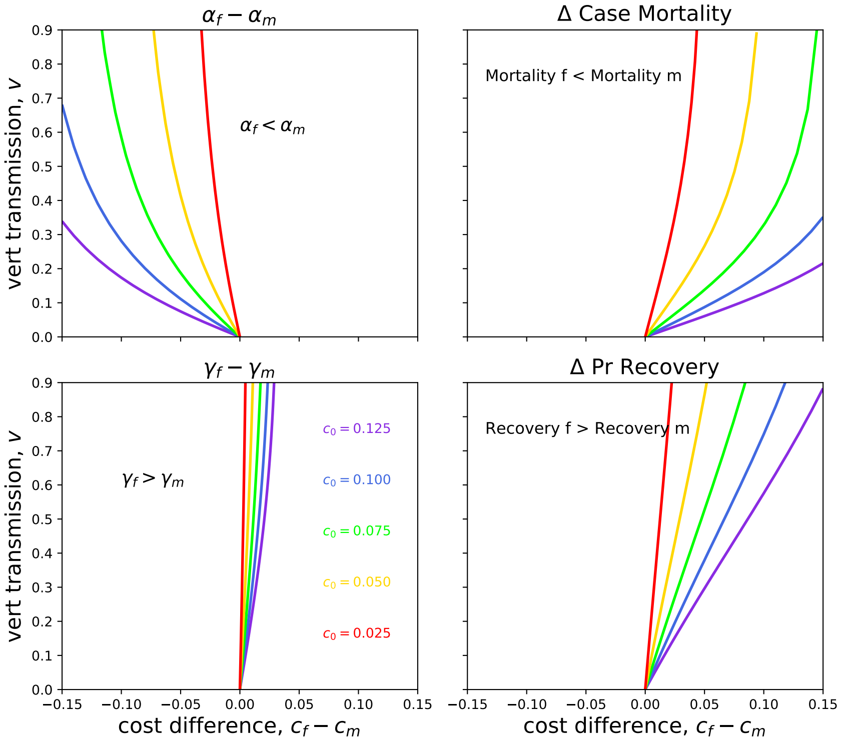

Figure 11: The area encompassed by regions in which (i) virulence (defined as either disease-related mortality or case mortality) is greater in males *and* (ii) recovery (defined as either recovery rate or probability of recovery) is greater in females grows when, all else being equal, the average cost of immune fucntion (defined as $c_0 = (c_f + c_m)/2$) increases (from red to violet curves). Results are based on $b_{max} = 2$, $\mu = 0.1$, $\beta_{\max} = 1.1$ and $d = 2$.

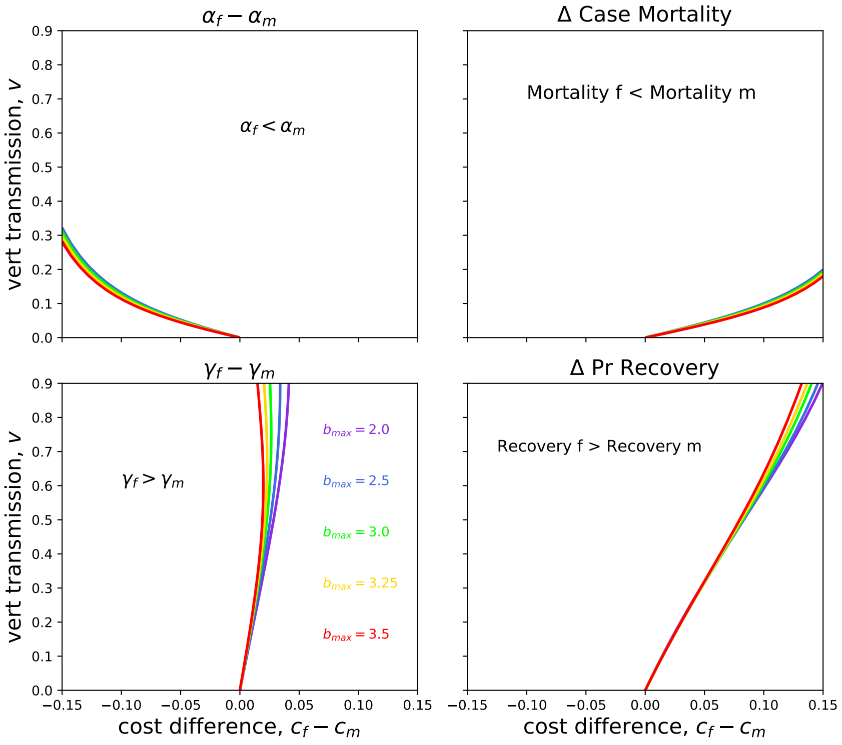

Figure 12: The area encompassed by regions in which (i) virulence (defined as either disease-related mortality or case mortality) is greater in males *and* (ii) recovery (defined as either recovery rate or probability of recovery) is greater in females grows when, all else being equal, maximum host fecundity b_{max} decreases (from red to violet curves). Results are based on average cost of immune fucntion (defined as $c_0 = (c_f + c_m)/2$) set to 0.1, $\mu = 0.1$, $\beta_{\text{max}} = 1.1$ and $d = 4$.

the face of lower risk of mortality is both a reasonably robust and sensible co-evolutionary prediction.

4 Extension to Immune Activation Costs Only

4.1 Preamble

In the model outlined above, we have assumed that the cost of immune function paid by the host is ongoing. In other words, the reduction in host fecundity due to γ_X is realized regardless of host disease status. In this section we modify the model presented in Section 3 of this supplement by imposing a fecundity cost only on those hosts who are infected with the disease. The modified model allows us to explore the consequences of costly adaptive immune response, rather than a costly innate response. Ultimately, we find that the qualitative patterns described in the main text are robust to this change in model assumptions.

4.2 Modified Model

It now becomes necessary for us to model the resident ecological dynamics using

$$\frac{dS_f}{dt} = \frac{S_f b_{ss} S_m + S_f b_{si} I_m + (1-v) I_f b_{is} S_m + (1-v) I_f b_{ii} I_m}{N} + \gamma_f I_f - S_f \beta_{ff} I_f - S_f \beta_{fm} I_m - \mu N S_f \tag{15a}$$

$$\frac{dS_m}{dt} = \frac{S_f b_{ss} S_m + S_f b_{si} I_m + (1-v) I_f b_{is} S_m + (1-v) I_f b_{ii} I_m}{N} + \gamma_m I_m - S_m \beta_{mf} I_f - S_m \beta_{mm} I_m - \mu N S_m \tag{15b}$$

$$\frac{dI_f}{dt} = \frac{v I_f b_{is} S_m + v I_f b_{ii} I_m}{N} + S_f \beta_{ff} I_f + S_f \beta_{fm} I_m - (\gamma_f + \alpha_f + \mu N) I_f \tag{15c}$$

$$\frac{dI_m}{dt} = \frac{v I_f b_{is} S_m + v I_f b_{ii} I_m}{N} + S_m \beta_{mf} I_f + S_m \beta_{mm} I_m - (\gamma_m + \alpha_m + \mu N) I_m \tag{15d}$$

where b_{si} reflects the fertility of a susceptible female and an infected male, b_{is} reflects the fertility of an infected female and susceptible male, and so on.

In the absence of the disease, the population again tends to a stable equilibrium size S_0 but this equilibrium size is now expressed as $\frac{b_{ss}}{4\mu}$. The stability of disease-free equilibrium

Table 1: Summary of predictions resulting from parameter sets chosen because they allow ESS values to be resolved over the same $(c_f - c_m, v)$-region illustrated in Figure 8 (but otherwise chosen arbitrarily). Columns labelled "A" present the fraction of $(c_f - c_m, v)$-region in which $\alpha_f^* < \alpha_m^*$ and $\gamma_f^* > \gamma_m^*$. Columns labelled "B" present the fraction of $(c_f - c_m, v)$-region in which the case fatality rate in males is higher and the case recovery rate in females is lower. Numbers in A and B are estimates by surveying a 25×25 grid of $(c_f - c_m, v)$-space. Table continued on next page.

$b_{\max}$	$\beta_{\max}$	d	A	B
2.000	0.750	0.100	0.333	0.451
2.000	0.750	0.150	0.328	0.486
2.000	0.750	0.200	0.326	0.512
2.000	1.000	0.100	0.326	0.434
2.000	1.000	0.150	0.320	0.453
2.000	1.000	0.200	0.318	0.488
2.000	1.100	3.500	0.483	0.731
2.000	1.100	3.750	0.501	0.733
2.000	1.100	4.000	0.515	0.738
2.000	1.100	4.250	0.528	0.739
2.000	1.100	4.500	0.539	0.747
2.000	1.250	0.100	0.320	0.419
2.000	1.250	0.150	0.315	0.438
2.000	1.250	0.200	0.310	0.466
2.000	1.500	0.100	0.315	0.410
2.000	1.500	0.150	0.310	0.427
2.000	1.500	0.200	0.310	0.443
2.000	1.750	0.100	0.317	0.400
2.000	1.750	0.150	0.310	0.418
2.000	2.000	0.100	0.310	0.398
2.000	2.000	0.150	0.306	0.410
2.000	2.250	0.100	0.317	0.389
2.000	2.250	0.150	0.304	0.403
2.000	2.500	0.100	0.310	0.386
2.000	2.500	0.150	0.306	0.397
2.000	3.000	0.100	0.309	0.378
2.000	3.000	0.150	0.307	0.389
2.000	1.600	3.500	0.406	0.730
2.000	1.600	3.750	0.418	0.734
2.000	1.600	4.000	0.429	0.739
2.000	1.600	4.250	0.437	0.741
2.000	2.100	2.500	0.322	0.715
2.000	2.100	2.750	0.333	0.722
2.000	2.100	3.000	0.346	0.726
2.000	2.100	3.250	0.355	0.728
2.000	2.100	3.500	0.365	0.738
2.000	2.600	0.500	0.282	0.502
2.000	2.600	0.750	0.267	0.592
2.000	3.100	0.250	0.294	0.411
2.000	3.100	0.500	0.286	0.488
4.000	0.750	0.100	0.346	0.395
4.000	0.750	0.200	0.346	0.411
4.000	0.750	0.300	0.352	0.432
4.000	0.750	0.400	0.355	0.451
4.000	0.750	0.500	0.362	0.472
4.000	1.000	0.100	0.336	0.386
4.000	1.000	0.200	0.334	0.398
4.000	1.000	0.300	0.336	0.414
4.000	1.000	0.400	0.339	0.424
4.000	1.000	0.500	0.339	0.443

b_{max}	β_{max}	d	A	B
4.000	1.250	0.100	0.330	0.376
4.000	1.250	0.200	0.326	0.386
4.000	1.250	0.300	0.325	0.402
4.000	1.250	0.400	0.328	0.414
4.000	1.250	0.500	0.330	0.426
6.000	0.250	0.100	0.403	0.443
6.000	0.250	0.200	0.414	0.470
6.000	0.250	0.300	0.424	0.494
6.000	0.250	0.400	0.437	0.507
6.000	0.250	0.500	0.450	0.514
6.000	0.500	0.100	0.362	0.400
6.000	0.500	0.200	0.365	0.416
6.000	0.500	0.300	0.370	0.432
6.000	0.500	0.400	0.374	0.443
6.000	0.500	0.500	0.381	0.458
6.000	0.750	0.100	0.344	0.384
6.000	0.750	0.200	0.342	0.394
6.000	0.750	0.300	0.346	0.405
6.000	0.750	0.400	0.349	0.416
6.000	0.750	0.500	0.354	0.424

b_{max}	β_{max}	d	A	B
8.000	0.125	0.100	0.459	0.499
8.000	0.125	0.200	0.470	0.509
8.000	0.125	0.300	0.478	0.538
8.000	0.125	0.400	0.475	0.528
8.000	0.125	0.500	0.488	0.550
8.000	0.250	0.100	0.397	0.434
8.000	0.250	0.200	0.406	0.450
8.000	0.250	0.300	0.416	0.474
8.000	0.250	0.400	0.421	0.490
8.000	0.250	0.500	0.429	0.501
8.000	0.500	0.100	0.357	0.395
8.000	0.500	0.200	0.360	0.406
8.000	0.500	0.300	0.365	0.418
8.000	0.500	0.400	0.368	0.424
8.000	0.500	0.500	0.370	0.434

solution to (15) now depends on the spectral radius of the positive matrix

$$\mathbf{K} = \begin{bmatrix} \frac{b_{is}v/2+S_0\beta_{ff}}{2S_0\mu+\alpha_f+\gamma_f} & \frac{S_0\beta_{fm}}{2S_0\mu+\alpha_m+\gamma_m} \\ \frac{b_{is}v/2+S_0\beta_{mf}}{2S_0\mu+\alpha_f+\gamma_f)} & \frac{S_0\beta_{mm}}{2S_0\mu+\alpha_m+\gamma_m} \end{bmatrix} \tag{16}$$

with the equilibrium changing from asymptotically stable to unstable as the spectral radius moves from less than to greater than 1. When the disease-free equilibrium is unstable we predict the existence of an endemic equilibrium, again denoted $(\bar{S}_f, \bar{S}_m, \bar{I}_f, \bar{I}_m)$, which can be determined computationally using the iterative method laid out in the Matlab function `ResEquilAct.m`.

Knowing the endemic equilibrium allows us to compute the invasion fitness of a mutant parasite strain as the spectral radius of

$$\tilde{\mathbf{K}} = \begin{bmatrix} \frac{v(b_{is}\bar{S}_m+b_{ii}\bar{I}_m)/\bar{N}+\bar{S}_f\tilde{\beta}_{ff}}{\gamma_f+\tilde{\alpha}_f+\mu\bar{N}} & \frac{\bar{S}_f\tilde{\beta}_{fm}}{\gamma_m+\tilde{\alpha}_m+\mu\bar{N}} \\ \frac{v(b_{is}\bar{S}_m+b_{ii}\bar{I}_m)/\bar{N}+\bar{S}_m\tilde{\beta}_{mf}}{\gamma_f+\tilde{\alpha}_f+\mu\bar{N}} & \frac{\bar{S}_m\tilde{\beta}_{mm}}{\gamma_m+\tilde{\alpha}_m+\mu\bar{N}} \end{bmatrix} \tag{17}$$

which we do in Matlab using the function `WpAct.m`.

The endemic equilibrium also allows us to compute the invasion fitness of a mutant strain of host as the spectral radius of

$$\hat{\mathbf{K}} = \mathbf{F}\mathbf{V}^{-1}. \tag{18}$$

As in the previous version of the model, the matrix $\mathbf{F}$, defined as

$$\frac{1}{2}\begin{bmatrix} \frac{b_{ss}(\hat{\gamma}_f,\gamma_m)\bar{S}_m+b_{si}(\hat{\gamma}_f,\gamma_m)\bar{I}_m}{\bar{N}} & \frac{\bar{S}_f b_{ss}(\gamma_f,\hat{\gamma}_m)+(1-v)\bar{I}_f b_{is}(\gamma_f,\hat{\gamma}_m)}{\bar{N}} & \frac{(1-v)(b_{is}(\hat{\gamma}_f,\gamma_m)\bar{S}_m+b_{ii}(\hat{\gamma}_f,\gamma_m)\bar{I}_m)}{\bar{N}} & \frac{\bar{S}_f b_{si}(\gamma_f,\hat{\gamma}_m)+(1-v)\bar{I}_f b_{ii}(\gamma_f,\hat{\gamma}_m))}{\bar{N}} \\ \frac{b_{ss}(\hat{\gamma}_f,\gamma_m)\bar{S}_m+b_{si}(\hat{\gamma}_f,\gamma_m)\bar{I}_m}{\bar{N}} & \frac{\bar{S}_f b_{ss}(\gamma_f,\hat{\gamma}_m)+(1-v)\bar{I}_f b_{is}(\gamma_f,\hat{\gamma}_m)}{\bar{N}} & \frac{(1-v)(b_{is}(\hat{\gamma}_f,\gamma_m)\bar{S}_m+b_{ii}(\hat{\gamma}_f,\gamma_m)\bar{I}_m)}{\bar{N}} & \frac{\bar{S}_f b_{si}(\gamma_f,\hat{\gamma}_m)+(1-v)\bar{I}_f b_{ii}(\gamma_f,\hat{\gamma}_m))}{\bar{N}} \\ 0 & \frac{v\bar{I}_f b_{is}(\gamma_f,\hat{\gamma}_m)}{\bar{N}} & \frac{v(b_{is}(\hat{\gamma}_f,\gamma_m)\bar{S}_m+b_{ii}(\hat{\gamma}_f,\gamma_m)\bar{I}_m)}{\bar{N}} & \frac{v\bar{I}_f b_{ii}(\gamma_f,\hat{\gamma}_m)}{\bar{N}} \\ 0 & \frac{v\bar{I}_f b_{is}(\gamma_f,\hat{\gamma}_m)}{\bar{N}} & \frac{v(b_{is}(\hat{\gamma}_f,\gamma_m)\bar{S}_m+b_{ii}(\hat{\gamma}_f,\gamma_m)\bar{I}_m)}{\bar{N}} & \frac{v\bar{I}_f b_{ii}(\gamma_f,\hat{\gamma}_m)}{\bar{N}} \end{bmatrix}, \tag{19}$$

is used to store information about host birth rates (fecundity) and the other matrix,

$$\mathbf{V} = \begin{bmatrix} \beta_{ff}\bar{I}_f + \beta_{fm}\bar{I}_m + \mu\bar{N} & 0 & -\hat{\gamma}_f & 0 \\ 0 & \beta_{mf}\bar{I}_f + \beta_{mm}\bar{I}_m + \mu\bar{N} & 0 & -\hat{\gamma}_m \\ -\beta_{ff}\bar{I}_f - \beta_{fm}\bar{I}_m & 0 & \hat{\gamma}_f + \alpha_f + \mu\bar{N} & 0 \\ 0 & -\beta_{mf}\bar{I}_f - \beta_{mm}\bar{I}_m & 0 & \hat{\gamma}_m + \alpha_m + \mu\bar{N} \end{bmatrix} \tag{20}$$

is used to store information about loss rates from the different categories of mutant individuals. Note that the inverse of this second matrix, denoted $\mathbf{V}^{-1}$, is given by

$$\begin{bmatrix} (\mu\bar{N} + \alpha_f + \hat{\gamma}_f)\tau_f & 0 & \hat{\gamma}_f\tau_f & 0 \\ 0 & (\mu\bar{N} + \alpha_m + \hat{\gamma}_m)\tau_m & 0 & \hat{\gamma}_m\tau_m \\ (\beta_{ff}\bar{I}_f + \beta_{fm}\bar{I}_m)\tau_f & 0 & (\mu\bar{N} + \beta_{ff}\bar{I}_f + \beta_{fm}\bar{I}_m)\tau_f & 0 \\ 0 & (\beta_{mf}\bar{I}_f + \beta_{mm}\bar{I}_m)\tau_m & 0 & (\mu\bar{N} + \beta_{mf}\bar{I}_f + \beta_{mm}\bar{I}_m)\tau_m \end{bmatrix} \tag{21}$$

where

$$\tau_f = \frac{1}{\mu\bar{N}(\mu\bar{N} + \alpha_f + \hat{\gamma}_f) + (\mu\bar{N} + \alpha_f)(\beta_{ff}\bar{I}_f + \beta_{fm}\bar{I}_m)}$$
$$= \frac{1}{\mu\bar{N}\hat{\gamma}_f + (\mu\bar{N} + \alpha_f)(\mu\bar{N} + \beta_{ff}\bar{I}_f + \beta_{fm}\bar{I}_m)} \quad (22)$$

and

$$\tau_m = \frac{1}{\mu\bar{N}(\mu\bar{N} + \alpha_m + \hat{\gamma}_m) + (\mu\bar{N} + \alpha_m)(\beta_{mf}\bar{I}_f + \beta_{mm}\bar{I}_m)}$$
$$= \frac{1}{\mu\bar{N}\hat{\gamma}_m + (\mu\bar{N} + \alpha_m)(\mu\bar{N} + \beta_{mf}\bar{I}_f + \beta_{mm}\bar{I}_m)}. \quad (23)$$

The Matlab function we used to compute host invasion fitness in this case is `WhAct.m`.

We follow the same basic steps laid out in Algorithm 1 to arrive at an evolutionarily stable quadruple α_f^*, α_m^*, γ_f^*, γ_m^*. The implementation of Algorithm itself is differs slightly from that discussed in Section 3 of this supplement. The new implementation for this model extension is found in `maindemoAct.m` and relies on other custom functions (see Table 2).

Using a Matlab implementation reflected by the demo `maindemoAct.m`, we are able to recover co-evolutionary predictions that follow the same qualitative patterns outlined for the case in which immune cost is on-going—paid by susceptible and infecteds alike. In particular, we find:

- In the absence of vertical transmission, the parasite-induced mortality rate and the recovery rate are both greater in the sex with the less expensive immune response (Figure 13).

- When the immune response is equally costly for both sexes, increased vertical transmission drives lower rates of parasite-induced mortality in females and, in turn, lower female recovery rates (Figure 14).

- Sex-specific immune costs and vertical transmission can interact to produce a co-evolved outcome where females recover at a higher rate from less lethal infections, relative to males (Figures 15 and 16).

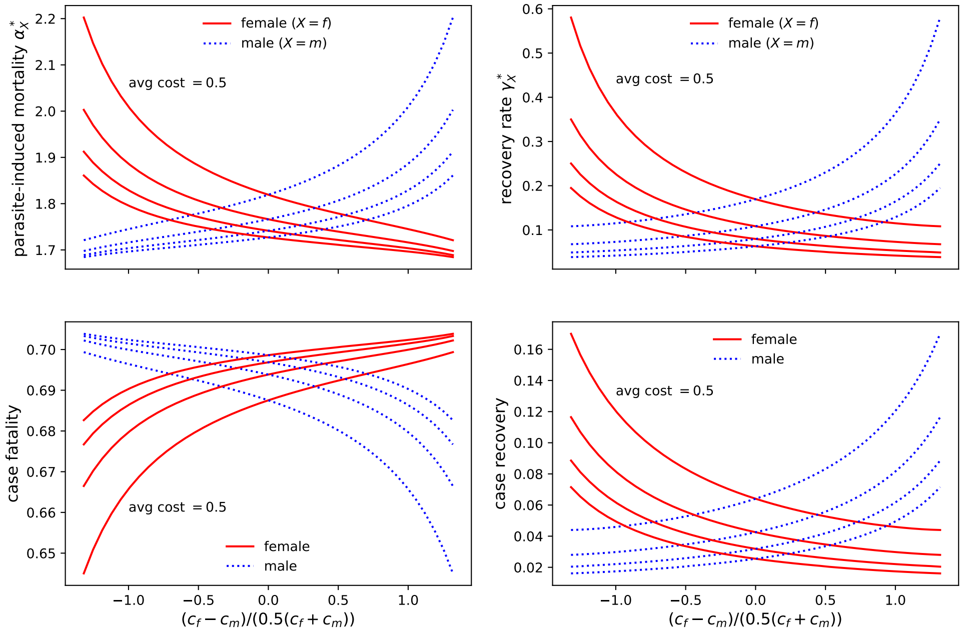

Figure 13: Predictions arising from a model with no vertical disease transmission ($v = 0$). Relative difference in cost of host immune function, measured as $(c_f - c_m)/(0.5(c_f + c_m))$ changes along the horizontal axis. Curves for average cost $0.5(c_f + c_m)$ from 0.5 to 1.25 (in steps of 0.25) are presented and vary continously as indicated. Additional parameters include $b_{\max} = 3.5$, $\mu = 0.1$, $\beta_{\max} = 3.1$, and $d = 4$ (for all X, Y). Predictions for females can be refelcted in the vertical line through the origin to obtain predictions for males.

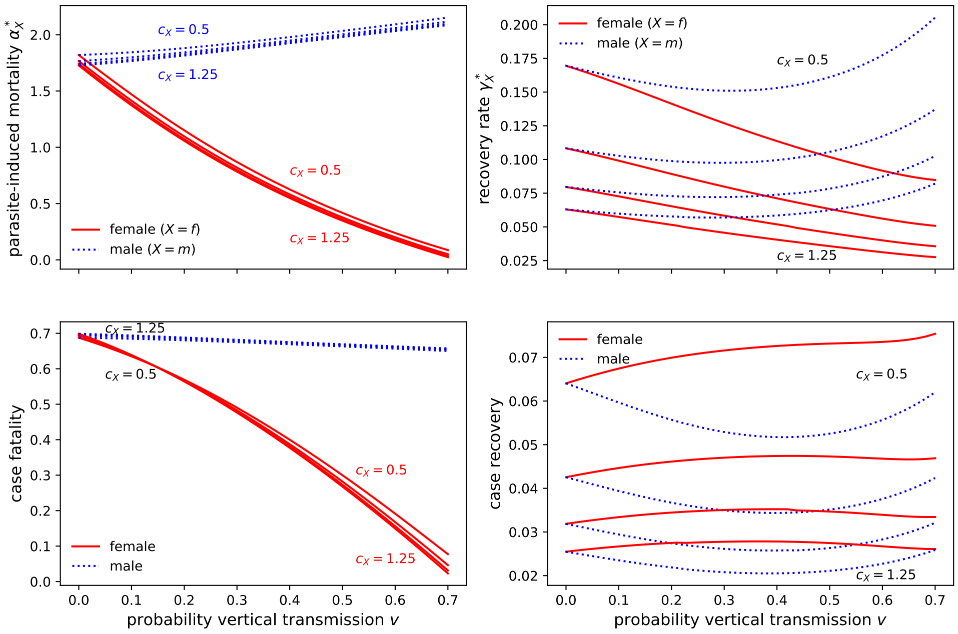

Figure 14: Predictions arising from a model with no sex difference in cost of immune function. Probability of vertical transmission, v, changes along the horizontal axis. Curves for different cost values $c_f = c_m = c_X$ are presented and vary from 0.5 (marked) to 1.25 in steps of size 0.25 as indicated. Female (red) and male (blue) curves meet at $v = 0$. Note that the relative order of female (red) case-fatality curves in the bottom left panel becomes reversed as one moves left to right. Additional parameters include $b_{\max} = 3.5$, $\mu = 0.1$, $\beta_{\max} = 3.1$, and $d = 4$ (for all X, Y).

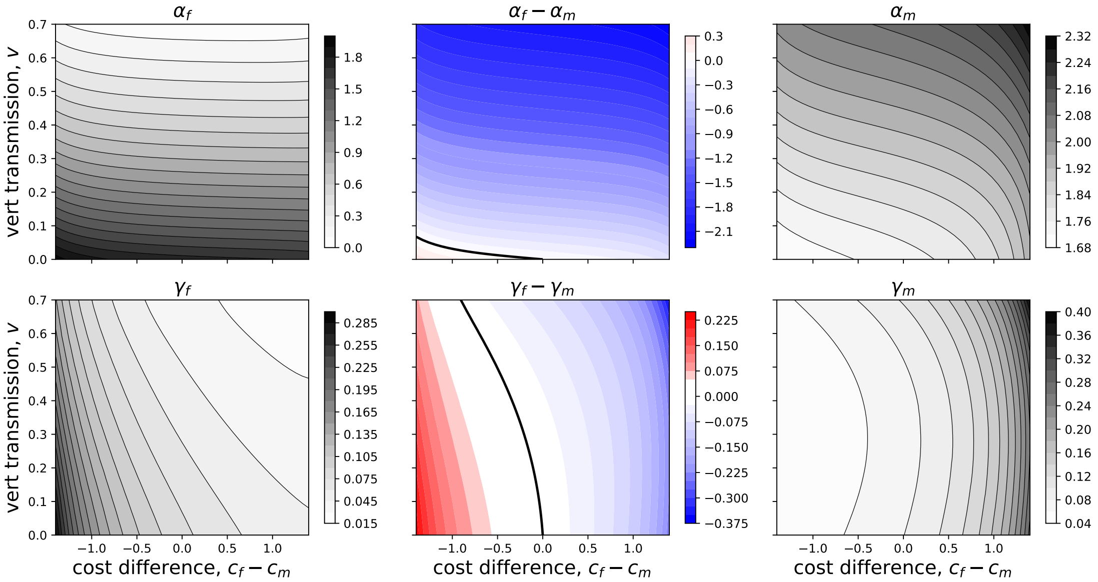

Figure 15: Changes in ESS predictions as vertical transmission and difference in the cost of immune function changes. Here, the cost of immune function is only paid by individuals infected by the pathogen. Center panels allow quick comparison of outcomes for the sexes, with red areas corresponding to greater values in females and blue areas corresponding to greater values in males. Results are based on $b_{max} = 3.5$, $\mu = 0.1$, $0.5(c_f + c_m) = 1$, $\beta_{\max} = 3.1$, and $d = 4$ (for all X, Y). Black curves in centre panels correspond to zero differences between the sexes and separate red regions from blue ones.

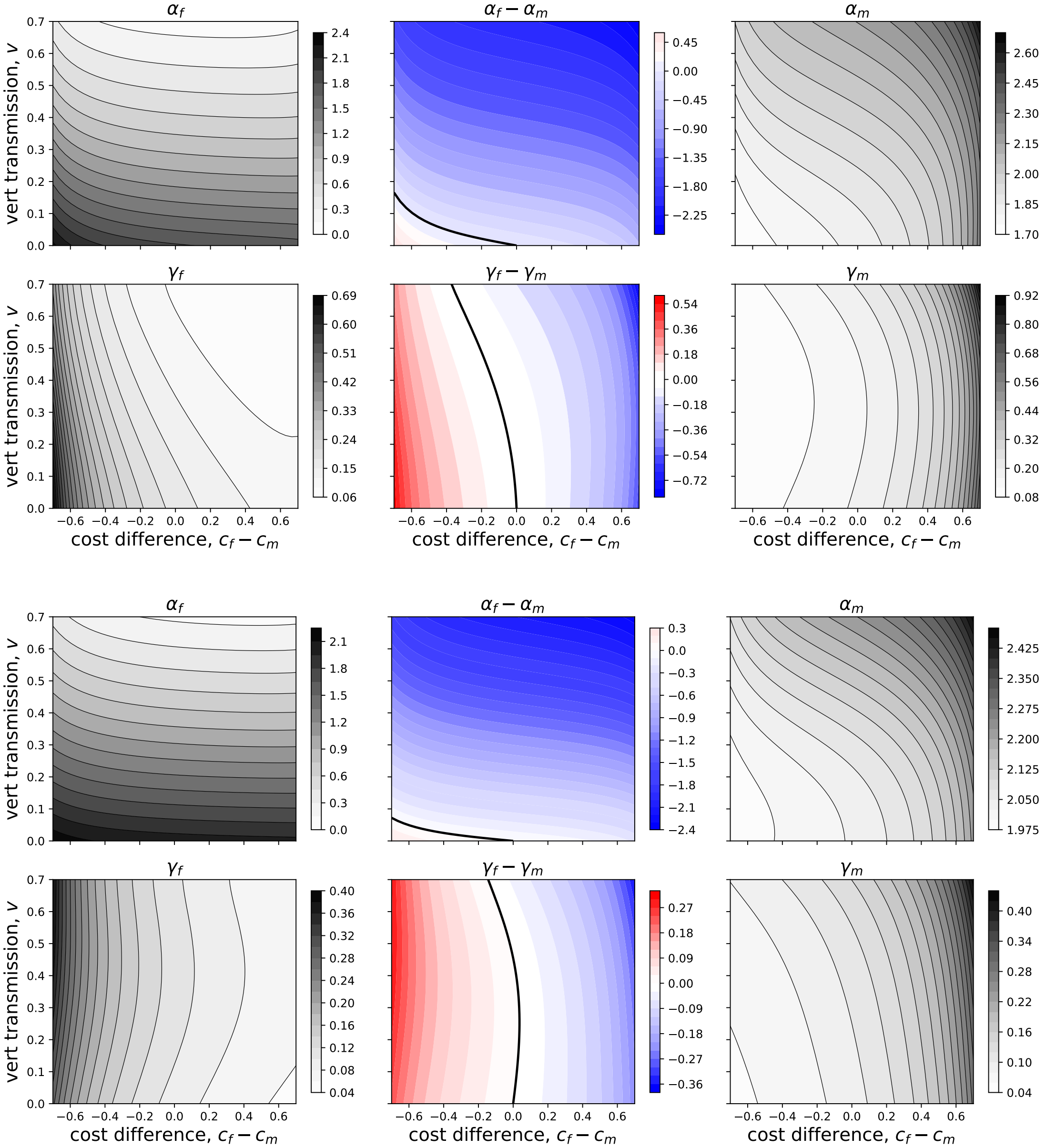

Figure 16: Comparison of predictions of the model extension explored in Section 5 (top panels) and the version of the model in Section 3 (bottom panels). Changes in ESS predictions as vertical transmission and difference in the cost of immune function changes. Here, the cost of immune function is only paid by individuals infected by the pathogen. Center panels allow quick comparison of outcomes for the sexes, with red areas corresponding to greater values in females and blue areas corresponding to greater values in males. Results are based on $b_{max} = 3.5$, $\mu = 0.1$, $0.5(c_f + c_m) = 0.5$, $\beta_{\max} = 3.1$, and $d = 4$ (for all X, Y). Black curves in centre panels correspond to zero differences between the sexes and separate red regions from blue ones.

5 Summary of Custom Code Used in Model Analysis

The code used in model analysis is available at https://github.com/evanjmitchell/on-maternity-and-immunocompetence and https://doi.org/10.5281/zenodo.6946414. A tabulated summary is provided in Table 2.

6 Data Availability

All numerical data generated for figures presented in this Supplement can be accessed at https://doi.org/10.5281/zenodo.6946414.

Table 2: Summary of custom code used in model analysis. Files can be accessed at https://github.com/evanjmitchell/on-maternity-and-immunocompetence. Code was implemented use core Matlab[6] version 9.6 (2019a release).

File	Description
**Model Presented in Main Text and Section 3 Above**	
`ResEquil.m`	Matlab function that finds an asymptotically stable equilibrium solution to system (2) iteratively. Takes model parameters as inputs and returns an equilibrium.
`Wp.m`	Matlab function that calculates invasion fitness of a mutant pathogen as the spectral radius of the matrix in equation (7). Takes model parameters and an estimate of the equilibrium state of the resident population as inputs and returns invasion fitness of pathogen.
`Wh.m`	Matlab function that calculates invasion fitness of a mutant pathogen as the spectral radius of the matrix in equation (14). Takes model parameters and an estimate of the equilibrium state of the resident population as inputs and returns invasion fitness of host.
`maindemo.m`	Demo Matlab script that illustrates how we estimate the joint ESS quadruple α_f^*, α_m^*, γ_f^*, γ_m^* following Algorithm 1. Script also assesses evolutionary stability by checking second-order conditions. The demo relies on functions `ResEquil.m`, `Wp.m`, and `Wh.m` and so all files should be saved to the same working directory. The output of the script is written to a comma-delimited file `maindemo.csv` and can be generated in about 0.5 sec on a standard laptop.
**Extension to Immune Activation Costs Only, Section 4**	
`*Act.m`	Analogues to functions `*Act.m` from the model featured in the main text and Section 3 (e.g. `maindemoAct.m` is analogous to `maindemo.m`). All files `*Act.m` should be saved to the same working directory. The output of the script is written to a comma-delimited file `maindemoAct.csv` and can also be generated in about 0.5 sec on a standard laptop.

References

1. Etheridge, A. *Some Mathematical Models from Population Genetics*. Lecture Notes in Mathematics (Springer, Berlin Heidelberg, 2012).

2. Berec, L. Mate search and mate-finding Allee effect: on modeling mating in sex-structured population models. *Theor. Ecol.* **11**, 225–244 (2018).

3. Caswell, H. & Weeks, D. E. Two-sex models: chaos, extinction, and other dynamic consequences of sex. *Am. Nat.* **128**, 707–735 (1986).

4. Úbeda, F. & Jansen, V. A. The evolution of sex-specific virulence in infectious diseases. *Nature Communications* **7**, ncomms13849 (2016).

5. van den Driessche, P. & Watmough, J. Further notes on the basic reproduction number. In *Mathematical Epidemiology*, Lecture Notes in Mathematics, pp. 159–178 (Springer, Berlin Heidelberg, 2008).

6. MATLAB. *Version 9.6.0 (R2019a)* (The MathWorks Inc., Natick, Massachusetts, 2019).

7. Alizon, S., Hurford, A., Mideo, N. & Van Baalen, M. Virulence evolution and the trade-off hypothesis: history, current state of affairs and the future. *J. Evol. Biol.* **22**, 245–259 (2009).

8. Cressler, C., McLeod, D., Rozins, C., van den Hooegen, J. & Day, T. The adaptive evolution of virulence: a review of theoretical predictions and empirical tests. *Parasitology* **143**, 915–930 (2016).

9. Acevedo, M., Dillemuth, F., Flick, A., Faldyn, M. & Elderd, B. Virulence-driven trade-offs in disease transmission: a meta-analysis. *Evolution* **73**, 636–647 (2019).

10. Hurford, A., Cownden, D. & Day, T. Next-generation tools for evolutionary invasion analysis. *Journal of the Royal Society Interface* **7**, 561–571 (2010).

11. Dercole, F. & Rinaldi, S. *Analysis of Evolutionary Processes: The Adaptive Dynamics Approach and Its Applications* (Princeton University Press, Princeton, New Jersey, 2008).

12. Beagley, K. W. & Gockel, C. M. Regulation of innate and adaptive immunity by the

female sex hormones oestradiol and progesterone. *FEMS Immunology and Medical Microbiology* **38**, 13–22 (2003).

13. Tan, I. J., Peeva, E. & Zandman-Goddard, G. Hormonal modulation of the immune system - A spotlight on the role of progestogens. *Autoimmunity Reviews* **14**, 536–542 (2015).

14. Christiansen, F. B. On conditions for evolutionary stability for a continuously varying character. *American Naturalist* **138**, 37–50 (1991).

15. Day, T. & Taylor, P. D. Evolutionary dynamics and stability in discrete and continuous games. *Evolutionary Ecology Research* **5**, 605–613 (2003).

16. Day, T. On the evolution of virulence and the relationship between various measures of mortality. *Proceedings of the Royal Society, B* **269**, 1317–1323 (2002).

17. Day, T. & Burns, J. G. A consideration of patterns of virulence arising from host-parasite coevolution. *Evolution* **57**, 671–676 (2003).
